# Reversing Blood Flows Act through *klf2a* to Ensure Normal Valvulogenesis in the Developing Heart

**DOI:** 10.1371/journal.pbio.1000246

**Published:** 2009-11-17

**Authors:** Julien Vermot, Arian S. Forouhar, Michael Liebling, David Wu, Diane Plummer, Morteza Gharib, Scott E. Fraser

**Affiliations:** 1 Biological Imaging Center, Beckman Institute, California Institute of Technology, Pasadena, California, United States of America; 2 Option in Bioengineering, California Institute of Technology, Pasadena, California, United States of America; 3 Electrical and Computer Engineering, University of California Santa Barbara, Santa Barbara, California, United States of America; Osaka University, Japan

## Abstract

Heart valve anomalies are some of the most common congenital heart defects, yet neither the genetic nor the epigenetic forces guiding heart valve development are well understood. When functioning normally, mature heart valves prevent intracardiac retrograde blood flow; before valves develop, there is considerable regurgitation, resulting in reversing (or oscillatory) flows between the atrium and ventricle. As reversing flows are particularly strong stimuli to endothelial cells in culture, an attractive hypothesis is that heart valves form as a developmental response to retrograde blood flows through the maturing heart. Here, we exploit the relationship between oscillatory flow and heart rate to manipulate the amount of retrograde flow in the atrioventricular (AV) canal before and during valvulogenesis, and find that this leads to arrested valve growth. Using this manipulation, we determined that *klf2a* is normally expressed in the valve precursors in response to reversing flows, and is dramatically reduced by treatments that decrease such flows. Experimentally knocking down the expression of this shear-responsive gene with morpholine antisense oligonucleotides (MOs) results in dysfunctional valves. Thus, *klf2a* expression appears to be necessary for normal valve formation. This, together with its dependence on intracardiac hemodynamic forces, makes *klf2a* expression an early and reliable indicator of proper valve development. Together, these results demonstrate a critical role for reversing flows during valvulogenesis and show how relatively subtle perturbations of normal hemodynamic patterns can lead to both major alterations in gene expression and severe valve dysgenesis.

## Abstract 2

The directionality of local blood flow in the zebrafish embryonic heart is essential for proper heart valve formation.

## Author Summary

The growth and development of vertebrates are critically dependent on efficient cardiac output to drive blood circulation. An essential step of heart development is the formation of heart valves, whose leaflets are made through a complex set of cellular rearrangements of endothelial cells. Endothelial cells experience high flow forces as blood circulates. Moreover, heart valves and associated structures can be malformed when flow forces are abnormal, suggesting that these flow forces are in fact required for proper valve formation. Whether it is the force of the blood flow, its directionality (forward or reverse), or both that are important is not clear. We studied the interplay during valve development between key genes known to be involved in the process and epigenetic influences such as flow forces. Using zebrafish, whose optical clarity allows analyzing blood flow patterns at high resolution, we identified the presence of reversing flows specifically at the level of valve precursors. By manipulating blood flow patterns, we show that reversing flows are essential for valve morphogenesis. Specifically, we show that the expression of the gene *klf2a* depends on the presence of reversing flows and is required for valve development. We predict that by influencing levels of *klf2a*, reversing flows constitute an important stimulus controlling the appropriate biological responses of endothelial cells during valve formation.

## Introduction

Formation of valves is a critical step in the development of a functionally mature heart, yet little is known about the mechanisms that initiate valve formation in vivo. In vertebrates, valves form from the endothelial cell layer located at the border between the atrium and the ventricle [1]–[3]. In fish, this region is called atrioventricular (AV) canal [1],[4],[5] and defines the endothelial ring [6]. The expression of genes specific to this territory depends on the activity of molecules secreted in the subjacent AV myocardium and on an elaborate combination of signaling pathways between the two cell layers, including Wnt/β-catenin, bone morphogenetic protein (BMP), and Notch signaling [4],[7]–[10]. Not surprisingly, aberrant patterning of the myocardial layer of the early heart can lead to valve defects, as the specification of the AV canal is impaired [6]. The analysis of zebrafish mutants has led to the identification of several cellular changes happening in the endothelial cell precursors during the process of valvulogenesis [1], and it has been shown that some of these changes are associated with physical stimuli provided by blood flow [11]. Interestingly, valve morphogenesis is clearly dependant on the geometry of the beating heart chambers, further suggesting that the physical environment near the developing valves plays a critical role for their development [11]. Along with previous observations demonstrating the importance of intracardiac fluid flow for cardiogenesis [4],[12]–[14], this offers the exciting possibility that the genetic programs that govern valve formation in vivo depend on intracardiac hemodynamics. Harvesting this possibility has been challenging, as some attempts to uncouple contractility and flow have been taken to suggest that they play opposing roles in modulating cell shape within the developing heart [12]; other studies have suggested that flow forces regulate looping, cell size and shape in the heart chambers, and the formation of trabeculae [5],[12]–[14]. A recent publication highlights the uncertainty concerning the role of flow during heart valve development, since it reports a cardiac contractility mutant that can form normal valves [15]; thus, something more than the mere presence (or absence) of flow or contractility must be involved in directing valve development.

The predominant model to explain endothelial cell response to flow envisions that the shear stress, which directly depends on the viscosity and the velocity of the blood, is the main physical stimulus. More recently, *disturbed flow* has been used as a general term to group abnormal flow patterns (including low flow, oscillatory flow, flow separation, gradients, turbulence, and reversing flows), potentially leading to atherogenic stimulus for endothelial cells [16]–[18]. This hypothesis is indirectly supported by observation that, in vitro, endothelial cells can be responsive to disturbed flows [19],[20], leading to an atherogenic-like cell response [21]. Thus, an attractive hypothesis is that heart valves form as a developmental response to disturbed blood flows. A key prediction from this model is that altering flow patterns within the heartbeat cycle should directly affect valvulogenesis. In vitro approaches have so far been unsuccessful in addressing this question, possibly due to the absence of specific valve markers usable in vitro and to the difficulty to mimic in vitro the complexity of flow patterns observed in vivo.

To circumvent these limitations, we characterized embryonic zebrafish heart flow in vivo to identify a critical feature of the flow pattern associated with valve specification and tested its importance using a set of experimental manipulations including both genetic and pharmacological approaches. Taking advantage of high-speed imaging, we quantified the flow patterns generated in the beating heart and compared them with anatomical landmarks of the heart specified by expression patterns of known genes. Using antisense morpholine oligonucleotides (MOs) and drugs to alter these flow patterns in zebrafish, we show that reversing flow is essential to trigger flow-responsive genes in the AV canal and for initiating valvulogenesis. Our findings validate a key prediction of a specific and local role for reversing flows during cardiogenesis.

## Results

### Reversing Flows Are Higher in the AV Canal

**Figure 1:**
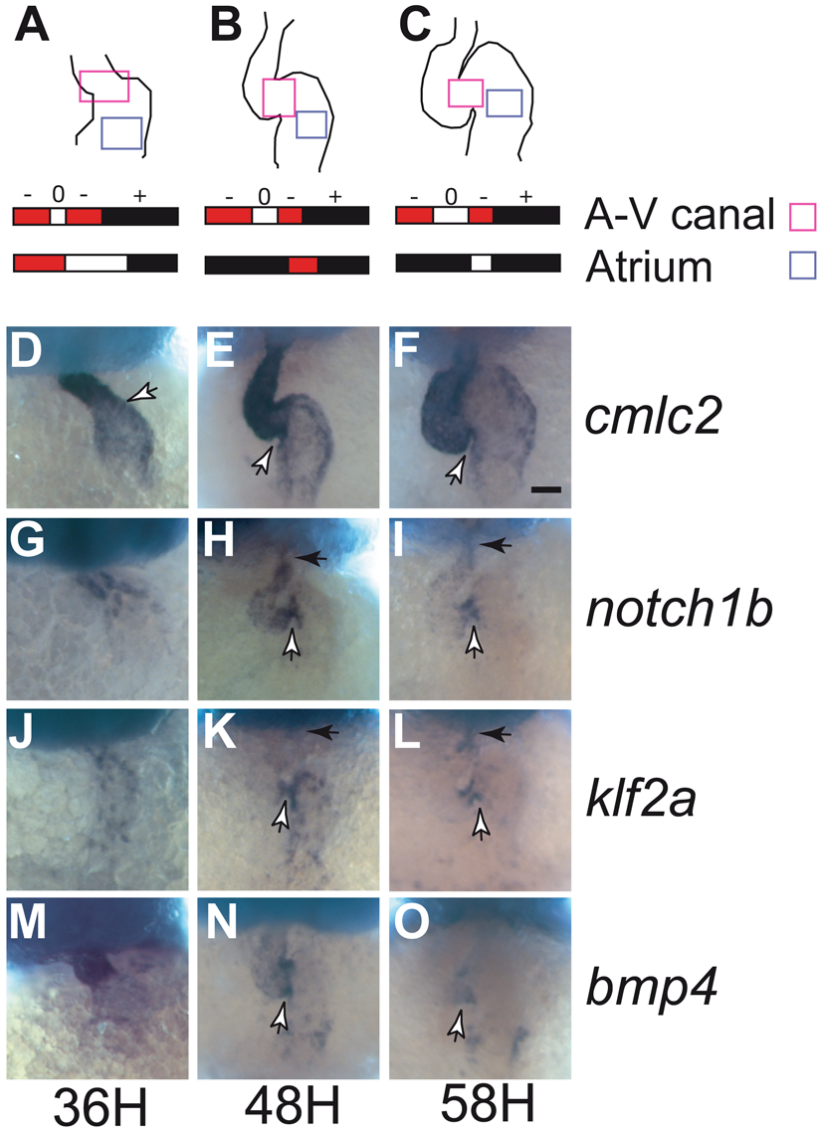
Transvalvular oscillatory flow patterns change during heart valve morphogenesis and gene expression in the AV canal. (A–C) Average transvalvular flow direction as a function of time for wild-type hearts as seen in the AV canal (light magenta box) or atrium (light blue box) between 36, 48, and 58 hpf in the area highlighted in the heart drawings (ventral view, anterior to the top). Anterograde flow from the atrium to ventricle is shown in black, retrograde flow from the ventricle to the atrium in red, and no flow between the chambers is shown in white. The sequence of time segments with retrograde, anterograde, and no-flow fractional periods are depicted in red, black, and white, respectively. The retrograde flow fraction (RFF) is the fraction of the cardiac cycle that is red. (D–F) *cmlc2* expression reveals changes in heart morphology. *cmlc2* is expressed in the heart tube in the anterior region at 36 hpf (D), and is expressed strongly in the ventricle and weakly in the atrium at 48 and 54 hpf (E and F). (G–O) Expression of *notch1b*, *klf2a*, and *bmp4* progressively becomes localized to the AV canal during valve specification. mRNA distribution of *notch1b* (G–I), *klf2a* (J–L), *bmp4* (M–O) at 36 hpf (D, G, J, and M), 48 hpf (E, H, K, and N), and 56 hpf (F, I, L, and O). *notch1b* is found in the anterior part of the heart tube at 36 hpf (G), and becomes stronger in the AV canal and in the ventricle at 48 hpf (H). At 54 hpf, *notch1b* expression becomes restricted in the AV canal and the outflow tract (I). *klf2a* expression is found throughout the heart tube at 36 hpf (J) and becomes stronger in the AV canal and in the atrium at 48 hpf (K). At 56 hpf, *klf2a* is exclusively expressed in the AV canal and the outflow tract, displaying an expression pattern very similar to *notch1b* (L). *bmp4* expression is found in the anterior part of the heart tube at 36 hpf (M) and becomes progressively concentrated at the level of the AV canal from 48 to 54 hpf (N and O). Anterior to the top, white arrows point to the AV canal, black arrows to the outflow tract. Scale bar indicates 50 µm.

In order to better understand the roles played by blood flow in heart and valve development, we have developed imaging techniques to capture cardiac motion and analyze blood flow. Imaging with these tools reveals dramatic changes in intracardiac blood flow patterns during cardiac development: as the heart enlarges, blood flow becomes increasingly bidirectional until the stage at which functional valve leaflets emerge at the boundary between the atrium and the ventricle (Figure S1, Video S1, and unpublished data, see also [11],[22]). Although reversing blood flows are at times visible in the atrium and ventricle, reversing flows are most pronounced at the AV canal in the second and third days of development (Figure 1A–1C, Video S2). We quantified the degree of reversing flow by measuring the fraction of the cardiac cycle during which there is retrograde flow, and term this the retrograde flow fraction (RFF). RFF is largest at the AV canal at embryonic stages that precede valve formation. Our ability to observe intracardiac blood flow simultaneously with heart pumping dynamics and morphogenetic changes provides a direct means to assess the proposal that the presence of particular patterns of intracardiac blood flow play a critical role in heart valve development.

To better understand how reversing flow relates to valve development at the molecular level, we analyzed the expression pattern by in situ hybridization (ISH) of three known shear-related genes at the AV canal: *notch1b*, a zebrafish Notch homolog [23]–[25], *klf2a*, a transcription factor from the Kruppel-like factor (Klf) family [26], and *bmp4*, a secreted growth factor of the bone morphogenetic protein (Bmp) family [27]. Notch is essential for valve formation [28], and the Notch pathway is activated by shear stress in HUVEC cells [29]–[31]. *klf2a* and *bmp4* are expressed in the zebrafish conduction system [4],[21],[25],. Our analysis concentrated on the AV canal during its specification (between 22 and 48 hours postfertilization [hpf]) [4],[25] as well as slightly before valve leaflet formation (58 hpf) [11] (Figure 1G–1O). Both *notch1b* and *klf2a* were expressed in the endothelium (Figure 1G–1L; Figure S2, Video S3). In contrast, *bmp4* was expressed in the myocardium of the heart tube, starting around 20–22 hpf and later became restricted to the AV canal between 36 and 58 hpf (Figure 1M–1O). Strikingly, expression of *notch1b*, *klf2a*, and *bmp4* became restricted to the region of high reversing flow we identified in the AV canal as the heart matured.

### Reversing Flows Control Valve Morphogenesis in Addition to Shear Stress

**Figure 2:**
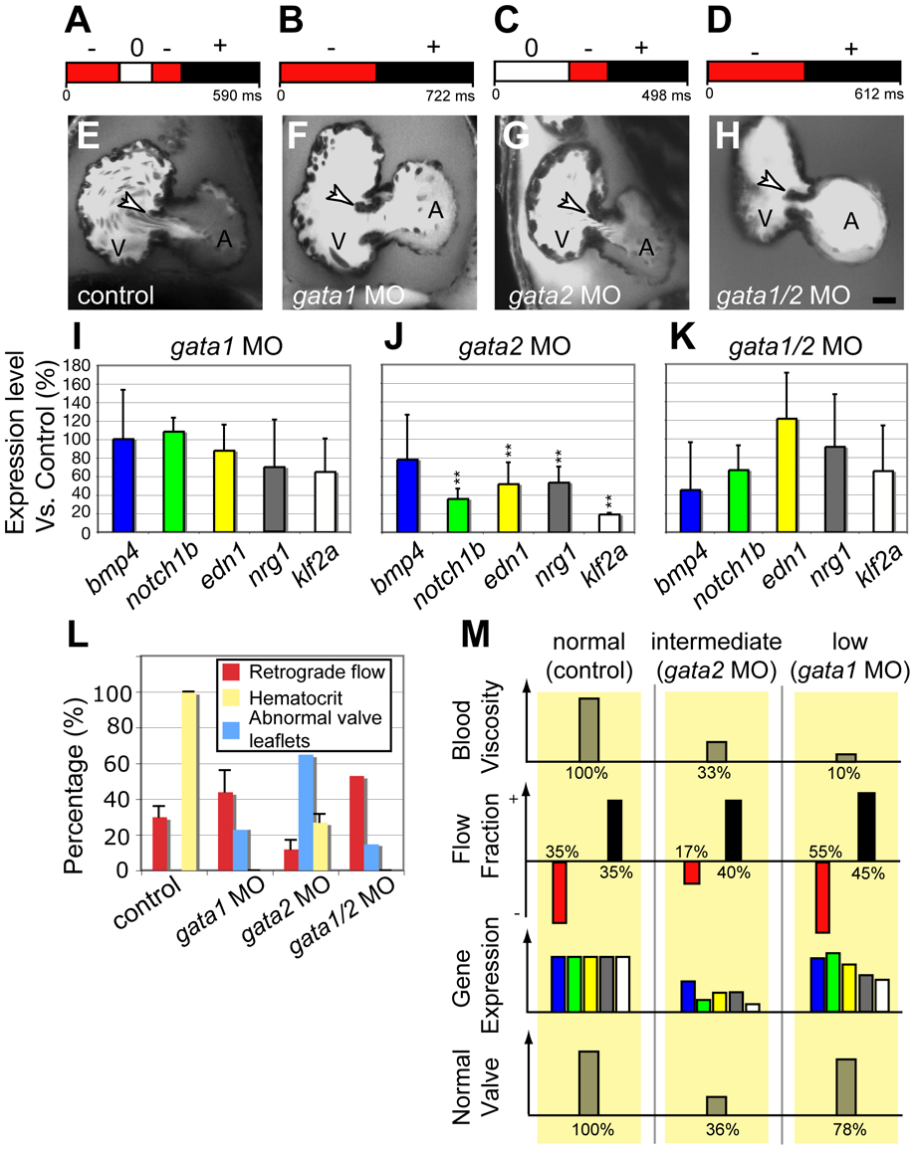
Decreased retrograde flow via lowered blood viscosity affects valve morphogenesis. (A–D) Flow pattern at 48 hpf in (A) control and after (B) *gata1*, (C) *gata2*, and (D) *gata1/2* knock down. *gata2* inactivation leads to a dramatic decrease in the RFF, whereas *gata1* and *gata1/2* knock downs exhibit increased RFF compared to the control. (E–H) Confocal sections of the valve-forming region in (E) control, (F) *gata1*, (G) *gata2*, and (H) *gata1/2* morphants. Only *gata2* morphants at 96 hpf show valve dysgenesis. Scale bar indicates 50 µm. (I–K) Quantitative RT-PCR showing the expression level of several flow-responsive genes after *gata1*, *gata2*, or *gata1/2* knock down. **p<0.01, ANOVA. (L) Percentage of embryos displaying valve malformation at 96 hpf (red bar), hematocrit level (yellow bar), and RFF (blue bar) observed in morphants and controls at 48 hpf. The proportions were significantly different at a 10% level of significance (α = 0.1). (M) Outline summarizing the experimental outcome of manipulating oscillatory flow by decreasing circulating blood cells. The color code for gene expression is the same as in (I).

In the developing zebrafish heart, where the Reynolds numbers are much less than one [14], flow patterns are dominated by the relationship between viscous forces and pressure gradients [32]. Thus, two methods of altering the reversing intracardiac blood flows in vivo are to: (1) manipulate blood viscosity, or (2) modulate pacemaker activity in order to change intracardiac pressure gradients [33]. To alter blood viscosity, we lowered the hematocrit by targeting two genes controlling early hematopoiesis in zebrafish, *gata1* and *gata2* [34], with MOs. Embryos injected with *gata1* MO are completely devoid of circulating blood cells [34], have a lower blood viscosity (reduced by ∼90%, see Materials and Methods), and display an increased RFF compared to controls (Video S4; ∼RFF: 45%±12% in *gata1* morphants, compared to 35%±7% in controls, Figure 2A and 2B). Embryos injected with *gata2* MO contain fewer circulating blood cells in comparison to wild-type embryos (72% fewer blood cells, Figure 2L, Video S5), have a reduced viscosity (∼70% lower than controls), and display a strongly reduced RFF (17%±4% of the heart cycle, Figure 2C; Video S4) in a majority of embryos (*n* = 13, 54%), highlighting the nonlinear relationship between heartbeat frequency and viscosity. When analyzed at 96 hpf, a majority of *gata1* embryos had normal valves (77% of embryos had normal valves, *n* = 13; Figure 2F and 2L); in contrast, the majority of *gata2* morphants displayed severe valve defects (64% of the embryos displayed abnormal valves, *n* = 14; Figure 2G and 2L). To make sure that the abnormal valve development was related to the lower RFF and not to other functions of *gata2*, we analyzed the effect of simultaneously inactivating *gata1* and *gata2*. This treatment further reduced blood viscosity, restored the RFF to 50% (Figure 2D, Video S4), and rescued valve formation (87% of embryos had normal valves, *n* = 8; Figure 2H and 2L). We also confirmed that lack of blood cells does not affect heart chamber patterning and vascular development (Figure S3). Because shear force depends directly upon viscosity, the reduced blood viscosity resulting from the *gata1* or *gata1/2* MOs reduces the magnitude of the shear forces throughout the cardiovascular system with respect to normal or *gata2* morphants (Figure S4). Thus, the normal valve development of the *gata1* and *gata1/2* morphants, and the abnormal valve development in the *gata2* morphants show that reversing flows, rather than magnitude of shear stress alone, are critical for valve leaflet formation (Figure 2M).

To better define the effects of RFF alteration in the *gata2* morphants, we used quantitative reverse transcriptase PCR (qRT-PCR) to study a set of flow-responsive genes. We compared expression of *bmp4*, *klf2a*, *notch1b*, *neuregulin1* (*nrg1*), and *endothelin1* (*edn1*) in wild-type, *gata1*, and *gata1/2* morphant embryos (Figure 2I–2K). Their expression levels in the *gata1* morphants remained close to the control baseline (Figure 2I), as did their levels in *gata1/2* morphants, except for a slight decrease in *bmp4* expression (about 2-fold, Figure 2K). In *gata2* morphants, two genes were significantly down-regulated: *klf2a* (about 5-fold reduction) and *notch1b* (about 2.5-fold reduction); *edn1* and *nrg1* display a mild reduction (about 1.5-fold reduction, Figure 2J).

Since wall shear stress (WSS) is a major stimulus for endothelial cell response in vitro, we explored whether it is also associated with the developmental changes we observe in vivo. Blood cell velocity measurements were used to estimate the WSS generated in the AV canal in control and altered flow conditions (summarized in Figure S4). In all *gata* morphants, the WSS is decreased due to the reduced blood viscosity. Interestingly, although *gata2* and *gata1* morphants display comparable amounts of WSS, they have opposite valve phenotypes. Thus, WSS magnitude cannot be the only determining factor for valvulogenesis. To explore this relationship further, we analyzed four heart contractility mutants (*cx36.7*, *myh6*, *ttna*, and *sih*; Figure S5A–S5E), and find that they have widely varying RFFs (Video S6). Furthermore, the mutants exhibiting a decreased RFF demonstrate both reduced *klf2a* expression (Figure S5F–S5H) and increased valve dysgenesis (Figure S5C, S5D, S5I, and S5J). Thus, results from animals with reduced blood viscosity and with reduced heart contractility suggest that, for normal development of valves, the reversing nature of the WSS is more important than its magnitude.

### *klf2a* Is Modulated by Low RFF in the AV Canal

**Figure 3:**
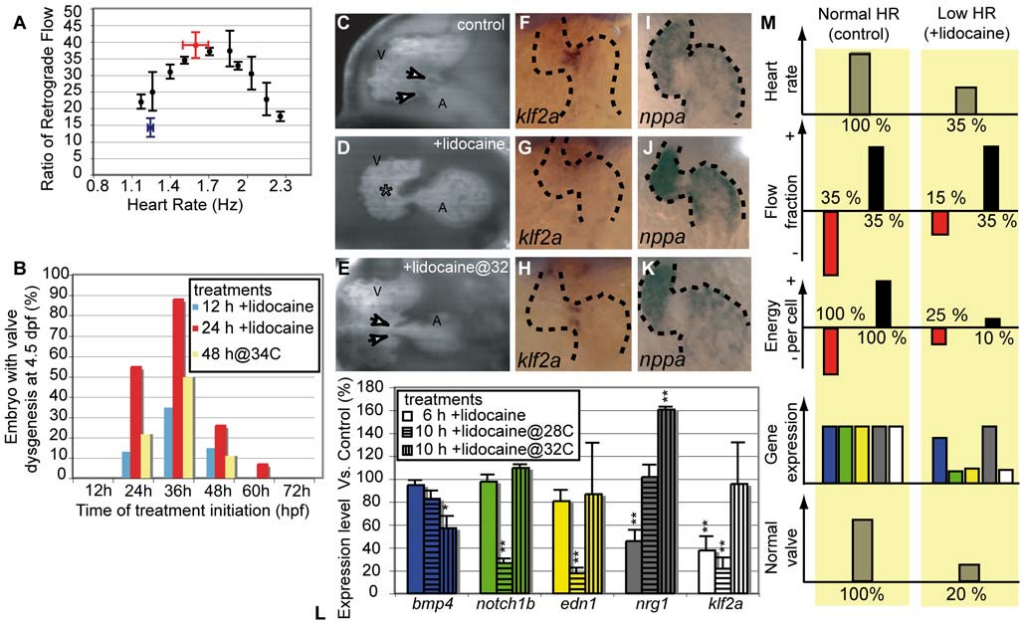
Decreased oscillatory flow decreases *klf2a* expression. (A) RFF is decreased by alterations in heart rate. The highest RFF is seen at the control heart rate (>30% between 1.5 and 2 Hz) at 48 hpf. Raising fish at lowered or elevated temperatures slows or speeds heart rate and significantly decreases RFF. Lidocaine treatment decreases heart rate and RFF (blue data point). The decreased heart rate and RFF is rescued by elevating the temperature to 34°C (red data point). (B) Decreased RFF from treatment with lidocaine or with high-temperature (34°C) leads to valve defects. The maximal effect is observed when treatment is initiated at 36 h. (C–E) Valve formation in normal and lidocaine treated embryos. (C) Embryos that were raised in control conditions have valve leaflets (white arrows). (D) Embryos in which RFF was decreased by lidocaine treatment from 31 to 55 hpf have endocardial tissue thickening (asterisk) but no valve leaflets are apparent (50%, *n* = 36). (E) Heart valve dysgenesis in fish exposed to 0.15% lidocaine for 24 h is rescued by incubating it at 34°C to restore normal RFF. Heart valve leaflets are present and function normally (white arrows). All embryos are imaged at 96 hpf. A, atrium; V, ventricle. (F–H) *klf2a* expression in 46-hpf-old embryos is altered by lidocaine treatment. (F) *klf2a* expression is localized at the AV boundary in control embryos. (G) *klf2a* expression decreases after 15-h lidocaine treatment (90%, *n* = 67). (H) Restoring heart rate and RFF to normal by raising the fish at 34°C restores *klf2a* expression (90%, *n* = 45). Anterior to the top. (I–K) *nppa* expression remains largely unaffected by lidocaine treatment and temperature rescue. (L) Quantitative RT-PCR showing the expression level of several flow-responsive genes after lidocaine treatment. *klf2a* expression is significantly decreased after 6 and 10 h of treatment and is restored by incubation at 34°C; 100% of expression corresponds to a normal expression level. *p<0.05; **p<0.01, ANOVA. (M) Outline summarizing the experimental outcome of decreasing oscillatory flow by decreasing heart rate. The color code for gene expression is the same as in (L).

We further explored the relationship between RFF and valve development by using lidocaine, a sodium channel blocker, to decrease heart rate [35], as well as increased temperature to increase heart rate [33]. Lidocaine increases the time from ventricular contraction to the atrial contraction of the next heartbeat, thus lengthening the period between the onset of the E wave (early diastolic filling due to ventricular suction) and A wave (ventricular filling due to atrial contraction). Slowing the heart rate by only 30% reduced the RFF by as much as 60% (Figure 3A). Similarly, warming the animal by 2–4°C sped up the heart and reduced the RFF (Video S7). Because lidocaine is easily applied and rinsed out, we could decrease the RFF for defined periods to find the stages at which oscillatory flow is critical for valve development. Starting at 24, 36, or 48 h of development, we incubated fish in lidocaine for either 12 or 24 h, after which the fish were returned to normal medium (Figure 3B). When scored at 96 hpf, valve leaflets were evident in all control embryos (no lidocaine exposure; Figure 3C; Video S8). In contrast, fish in which lidocaine reduced the RFF displayed a range of valve defects (Figure 3B, blue bars and red bars). Similar defects were observed after reducing the RFF with elevated temperature (Figure 3B, yellow bars). In the subtlest defect manifestations, valve leaflets did not form (Figure 3D; Figure S3B; Video S8). In more extreme cases, the heart retained an immature tubular shape (17%, *n* = 36; Figure S6). The defects cannot be from a side effect of the lidocaine, as slight warming of the animals to restore heart rate, and thereby RFF, to normal rescued valve leaflet formation (Figure 3E, 3H, and 3K, Video S8). The highest proportion of valve defects resulted when 12- or 24-h lidocaine treatments were initiated at 36 hpf, suggesting the greatest sensitivity to decreased RFF from 36–48 hpf (Figure 3B). Interestingly, this time window corresponds to the period when *bmp4*, *notch1b*, and *klf2a* normally become restricted to the AV canal.

To explore the timing relationships between the flow-responsive genes, we analyzed their expression by ISH after a 5- or 15-h lidocaine treatment, starting at 31 hpf. *klf2a* expression significantly decreased in as little as 5 h of treatment (Figure 3, compare 3F and 3G), indicating that *klf2a* may be an immediate target of the mechanism(s) that sense RFF. In contrast, expression of *notch1b* was normal after the short lidocaine treatment, but was decreased after 15 h of treatment (Figure S7). Quantitative PCR studies show that *klf2a*, *edn1*, and *notch1b* were strongly down-regulated after 10 h of lidocaine treatment, started at 36 h, (about 5-fold reduction compared to controls); whereas *nrg1* and *bmp4* expression levels were almost normal (Figure 3L and 3M). Shorter treatments (6 h) led to a significant decrease in *klf2a* and *nrg1* mRNA levels (about 2.5- and 2-fold reduction, respectively), suggesting that these two genes may be primary targets of retrograde flow (Figure 3L). The strong dependence of *klf2a* expression on the presence of oscillatory flow during both the 6- or 10-h treatments, as well as the similarity of its expression kinetics to those observed in cell culture [36], makes *klf2a* an excellent candidate as a key component in mediating the effects of oscillatory flow on valve specification, validating the proposed involvement of this gene in vertebrate cardiogenesis [37]–[39].

### *klf2a* Knock Down Affects Valvulogenesis

**Figure 4:**
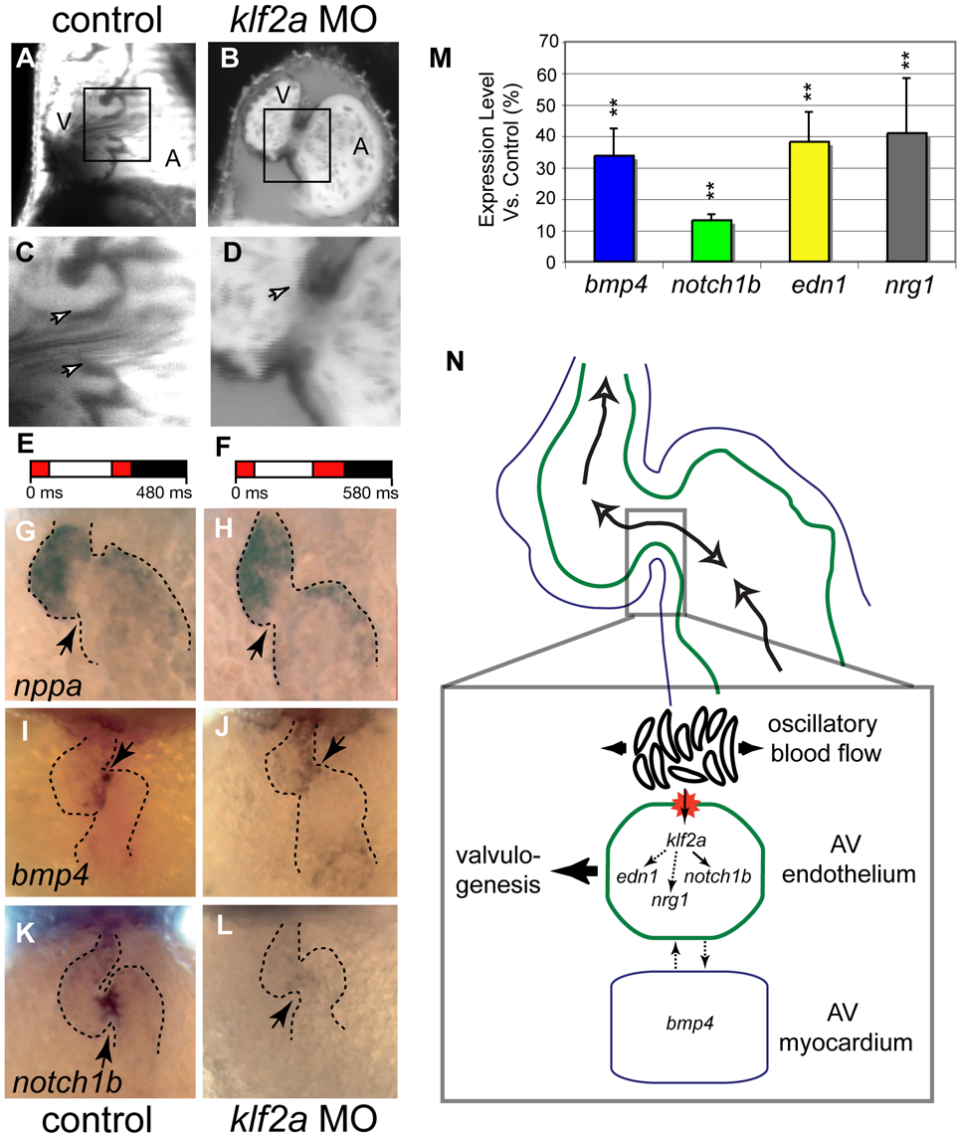
Morpholine antisense oligonucleotide treatment decreases expression of the flow-responsive gene *klf2a* results in valve dysgenesis. (A–D) Valve leaflets scored at 96 hpf show effects of *klf2a* MO. (A and C) Sham-injected embryos form normal heart valves. (B and D) *klf2a* MO-treated embryos display valve dysgenesis, often with a complete absence of valve leaflets. (C and D) Detailed views of valve morphology. (C) Control embryo has clearly distinguishable valve leaflets (arrows). (D) *klf2a* MO-treated embryo has no valve leaflets forming from the endocardium (arrow) (52%, *n* = 46). The proportions were significantly different at a level of significance α = 0.01. Scale bars indicate 50 µm. (E and F) Average flow pattern at 48 hpf in controls (E) and *klf2a* morphants (F) showing that the RFF is unaffected in the mutants but that the heart rate is slightly decreased. (G–L) Expression of three marker genes at 48 hpf in normal and *klf2a* morphants. (G–J) *nppa* expression is normal in the *klf2a* morphants, showing that chamber specification occurs independently of *klf2a*. (I and J) *bmp4* mRNA distribution at 48 hpf showing that expression is decreased in the MO-treated embryo in the AV node region at 48 hpf (*n* = 23, 40%; compare expression at arrow in panels [I and J]). (K and L) *notch1b* expression at 48 hpf decreases in the AV boundary of the *klf2a* morphants (*n* = 45, 71%). Arrows point to the AV boundary in all panels (G–L). (M) Summary of quantitative RT-PCR showing the expression level of flow-responsive genes in *klf2a* morphants. Expression of all genes decreases significantly, confirming the down-regulation of *bmp4* and *notch1b* observed by ISH. **p<0.01, ANOVA. (N) Summary diagram of *klf2a* function during heart valve formation. *klf2a* acts as a transcriptional relay between the reversing flow generated by the circulating blood cells at the AV canal and several genes activated in the AV endothelial cells (such as *notch1b*, *neuregulin1*, and *endothelin1*). *klf2a* also affects the expression of *bmp4*, revealing a possible interaction between myocardium and endothelium essential for valve morphogenesis.

We tested whether *klf2a* is required for valve formation by knocking down its expression using MOs, and obtained AV valve dysgenesis phenotypes that were remarkably similar to those of embryos exposed to reduced oscillatory shear stress (scored at 96 hpf; Figure 4A–4D); 52% of *klf2a* MO-treated embryos (*n* = 36) revealed valve dysgenesis; none of the sham- or control-injected embryos (*n* = 45) showed abnormal valve development (Figure 4A–4D). This similarity in phenotypes suggests that expression of *klf2a* is a key part of the genetic program that makes valve development responsive to normal oscillatory flow (Figure S8). In mouse, loss of *Klf2* is associated with heart failure and altered cardiac output [38]. In our studies, the zebrafish *klf2a* morphants displayed a heart rate similar to that of the control embryos at 48 hpf (1.7 Hz; Figure 4E and 4F), and had normal flow patterns within the AV canal (*n* = 5, Figure 4E and 4F). We found that atrial and ventricular fates are properly assigned in the *klf2a* morphants, because the chamber-specific expression of *nppa*, *bmp4*, and *cmlc2* appear normal (Figure 4G and 4H; Figure S9A–S9D). Thus, the effects of our MO experiments are not secondary to an alteration in heart structure or blood flow. ISH revealed that the first apparent molecular defects in the *klf2a* morphants are a decrease in *notch1b* expression at 36 hpf and a lack of *notch1b* expression at the AV boundary of the heart at 46 hpf (Figure 4L and Figure S9E and S9F), consistent with previous work showing that *klf2* lies upstream of Notch in HUVEC cells [40]. In contrast, *bmp4 *expression is normal at 36 hpf and slightly decreased at 46 hpf (Figure 4I and 4J and Figure S9C and S9D). When measured by qRT-PCR, the expression levels of *bmp4*, *edn1*, and *nrg1* were lower than normal by at least a factor of two (Figure 4M); the strong decrease in *notch1b* expression seen by ISH corresponds to a 10-fold reduction compared to controls (Figure 4M). Together with the fact that *klf2a* expression is a primary target of oscillatory flow, these data indicate that *klf2a* functions upstream of many known flow-induced genes in the process of AV valve formation in response to oscillatory flow.

### Cell Shape Is Affected by Decreased Reversing Flows during Valve Invagination

**Figure 5:**
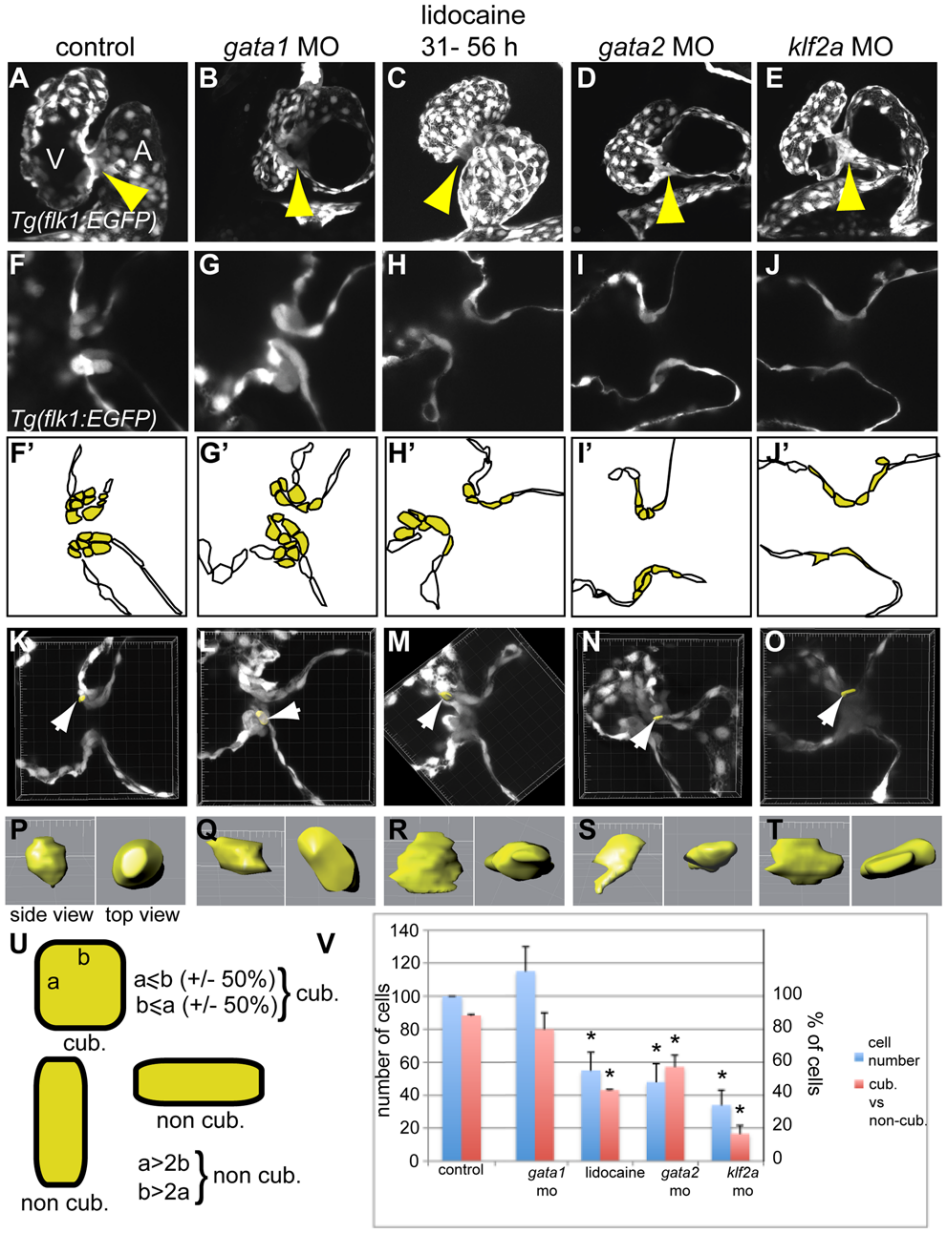
Comparison of the valve phenotype between the different treatments affecting valvulogenesis in transgenic *Tg(flk1:EGFP) * zebrafish at 72 hpf. GFP is expressed in the endothelial cell layer and highlights the developing valves. (A, F, and F') control embryo, (B, G, and G') *gata1* morphant, (C, H, and H') lidocaine treated, (D, I, and I') *gata2* morphant, and (E, J, and J') *klf2a* morphant. Each treatment lead to an incomplete ingression of the endothelial cells in order to make a functional leaflet except in *gata1* morphants. (F'–J') Schematic representation of the panels (F–J) underlining the endothelial cells within valve-forming region (yellow) and the heart lumen (white). A, atrium; V, ventricle. (K–O) Three-dimensional reconstruction of 10 µm depth of the AV area in control (K), gata1 morphants (L), lidocaine-treated embryo (M), and *gata2* (N) and *klf2a* (O) morphants. The white arrows point to the cell that has been reconstructed in three dimensions and which is presented in (P–T). (P–T) Side view (left) and top view (right) of a reconstructed cell of the AV canal. (U) Schematic drawing showing the approach used to define cuboidal versus non-cuboidal cell shape. (V) Graph summarizing the number of cells counted in the AV canal (corresponding to the yellow cells in [F'–J']) (blue bars), and the ratio between cuboidal versus non cuboidal cell shape (red bars). Yellow arrows in (A–E) point to the endocardial ring.

Zebrafish valves emerge from the endothelium through the combined actions of cell rearrangements and cell shape changes [1],[11]. To characterize the leaflet phenotype in the different mutants exhibiting altered RFF, we analyzed cell number and cell shape using the *Tg(flk1:gfp)* fish line [41] at 72 hpf, a stage at which the valve invagination is clearly visible [11]. In this line, the GFP accumulates in endothelial cells, but the fluorescence level is different from cell to cell. The inherent brightness variation allows us to count and assess the shape of every endothelial cell in the heart. Focusing our analysis on the AV canal, we found that the endothelial ring forms in every morphant and lidocaine-treated embryo (Figure 5A–5E). Strikingly, *gata1* and *gata1/2* morphants display normal cell numbers in the AV canal (Figure 5A and 5B and unpublished data); however, all treatments that disrupt the invagination of valve leaflets (*gata2* MO, *klf2a* MO, and lidocaine) exhibit decreased endothelial cell number in the AV canal compared to the controls. Three-dimensional volumetric measurement of the endothelial AV cells reveals that wild-type controls as well as the *gata1* and *gata1/2* morphants possess endothelial cells that are cuboidal (Figure 5A and 5B, 5F–5G, and 5Q; Video S9); in contrast, the endothelial cells remain flat and elongated in *gata2* morphants, *klf2a* morphants, and lidocaine-treated embryos (Figure 5C–5E, 5H–5J, and 5Q; Video S9). These differences precede the absence of valve invagination in embryos with decreased RFF and suggest that cell remodeling is important for leaflet morphogenesis. Taken together, our results show that the loss of *klf2a* expression, lack of invagination, decreased endothelial cell number, and abnormal endothelial cell shape characterize the effects of decreased RFF.

## Discussion

The beating heart is a highly dynamic structure. Its contraction generates multiple types of forces at different scales: Although both the myocardial and endocardial cells undergo a compression-stretching sequence during each contraction at the tissue scale, individual endothelial cells directly experience shear stress and oscillatory flows generated by moving blood. Although it is well established that both cell types are responsive to mechanical cues [17],[42]–[44], it has been difficult to clearly state which is the mechanical stimulus activating endothelial cells to respond to flow. To address this question, we have applied fast imaging on live embryos to carefully describe the flow patterns generated at the earliest stages of the valve development. Based on analysis of both live embryos and gene expression in fixed tissues, we find that the specific accumulation of *klf2a* transcripts within the valve precursor correlates with the presence of reversing flow in the AV canal and that altering flow patterns in the AV canal affects gene expression patterns in the endothelial cell layer. The differential response of endothelial cells to the presence or absence of reversing flows gives rise to an area prone for valvulogenesis. This response gets reinforced as reversing flows gradually concentrate in the AV canal. This phenomenon can be explained by the progressive reduction of the AV diameter as the atrium and ventricle loop, and the endothelial ring develops. Our results show that valvulogenesis results through the combination of a complex set of morphogenetic changes and are in full agreement with the different studies on the subject [5],[6],[11]–[14],[45].

### The Morphogenesis of Valve Leaflets Depends on Blood Flows in Zebrafish

In zebrafish, the first step of valvulogenesis involves the clustering of endothelial cells at the AV boundary. Cells coalesce to form an endothelial ring lining the AV canal between 24 and 48 hpf [6]. As seen previously [6], we found that flow is not necessary for endocardial ring formation. However, blood flow is critical for cell shape change and leaflet invagination. Knocking down *klf2a* does not affects endothelial ring formation, confirming that *klf2a* function starts when its expression becomes detectable in the AV canal. Our data together with those of others [11] show that the endothelial ring is assembled in a region coinciding with *klf2a* expression, and that reversing flows progressively increase in amplitude specifically at the AV canal after the endothelial ring forms. This timing suggests that the effects of blood flow act after an initial patterning that is guided by a genetic program, reminiscent of the process acting in vascular development [46]. Thus, it appears that the earliest steps of heart development can be considered as genetically hardwired, but that secondary events, such as valvulogenesis, are contingent on the presence of reversing flows.

Zebrafish valve development appears to be somewhat divergent from the process described in amniote vertebrates. In chicken and mice, valve leaflets arise from a mesenchymal cushion; in zebrafish, valves emerge directly through an invagination of the AV endothelium [11]. The origins of this morphogenetic process are unclear, but it allows the maturation of a functional valve in less than 96 h of embryonic development [11],[22]. Our results show that this morphogenetic mechanism is dependent on reversing flow forces. Interestingly, the absence of invagination correlates with a lack of cell shape change that would normally occur during this process. Many observations using endothelial cell culture have shown that the presence of flow activates signaling pathways implicated in cytoskeletal remodeling [17],[31]. It is thus tempting to speculate that reversing flows initiate the invagination process by stimulating the necessary movements and cytoskeletal rearrangements of endothelial cells in the AV canal to build a functional valve.

### Necessity and Modulation of *klf2a* Expression for Normal Valve Formation Highlights the Genetic Link between Biomechanical Stimulus and Cell Response to Reversing flows

The formation of heart valves allows unidirectional flow to be sustained as the peripheral vasculature develops and the increase in systemic resistance reduces the net flow of the valveless heart that results in the appearance of retrograde flow. The RFF is greater in the AV canal than in the rest of the heart or the cardiovascular system. The AV canal, a constriction, is exposed to high hemodynamic forces due to the higher velocities generated in areas with reduced cross section. Our studies clearly show that, although the drop in WSS magnitude affects gene expression levels in the heart, they are not sufficient to explain the abnormality in valve formation. Another aspect of the WSS, namely its oscillating directionality due to reversing flows, has to be included to understand the apparition of valve abnormalities in the *gata2* morphants and not in the *gata1* morphants where the shear forces are the lowest. Our quantitative imaging analysis strongly suggests that reversing flows are the proper stimulus controlling valve formation. Reversing flows have been observed in the developing cardiovascular system of many vertebrates ([47] and S. E. Fraser, unpublished data) and could be involved in other important steps of cardiovascular development. Among the many genes responsive to flow, *klf2a* seems to be specifically responsive to disturbed flows as observed both in vivo (this study) and in vitro [36]. Although, a direct involvement of *klf2* (the homolog of *klf2a* in mouse) during valve development remains to be uncovered in higher vertebrates, this study should stimulate investigation of subtler valve alterations in these mutants [38]. Genetic evidence also suggests that *klf2* has atheroprotective roles in adult mice [48] and humans [21], further suggesting that *klf2*, reversing flows, and cardiac physiology and development are tightly interconnected and that *klf2* could also be implicated in the flow response during these processes. *klf2a* stands out as a possible early indicator of defective valve development. Nevertheless, it is clear that a number of other genes are mediating the response of endothelial cells to flow and that more investigations will be required to isolate them all as well as determine their interconnections.

### Reversing Flows Constitute a Unique Physical Stimulus for Valve Development

Given that heart-pumping activity and blood content constantly change as the heart develops, a patterning mechanism based on flow sensing provides a very practical way to coordinate the timing of valve formation with the pumping efficiency of the heart. In the context of valvulogenesis, reversing flows constitute an efficient signal by providing specific stimuli that dynamically locate the valve forming area. This hypothesis is fully consistent with emerging models arising from studies addressing the role of biomechanical stimuli during embryogenesis, which suggest that extrinsic forces and intrinsic hardwired programs are interconnected into feedback loops [49],[50]. The advantage of such a mechanogenetic interplay is that it offers the opportunity for cells to locally adjust to the rapid environmental changes occurring in dynamic environments in conjunction with organizing centers [51]. In such systems, cells can directly react to the dynamics of the organ and can properly adapt at the single-cell level to organize as a coherently growing tissue. In conclusion, we have demonstrated that heart rate and blood viscosity can modulate the duration of oscillatory flow in vivo and have presented a set of useful methods to control hemodynamic forces during cardiogenesis. Together, these simple approaches offer powerful tools for predicting and potentially treating dysgenesis of cardiac valves and broaden the array of mechanisms to consider for explaining the origins of congenital cardiac malformation.

## Materials and Methods

### Confocal Imaging

The Zeiss LSM 510 was used to image *Tg(flk1:gfp)* and BODIPY-ceramide (Molecular Probes) stained embryos to visualize valve structure. Embryos were anesthetized prior to imaging in 0.0175% tricaine and placed in agarose wells. All images were taken with a 40×/1.1 LD C-Apochromat water immersion lens. For high-speed imaging, the Zeiss LSM 5 LIVE was used to image BODIPY-ceramide–stained embryos and to visualize valve formation and flow patterns. Images of 256×256 pixels were acquired at 151 frames per second.

### High-Speed Video Microscopy

Brightfield images were taken with a Basler A602f CMOS camera mounted on a home-built microscope equipped with an Olympus 0.5 NA 10× air objective coupled with a 300-mm focal length tube lens. Images were acquired at 216 frames per second.

### Transvalvular Flow Characterization

Transvalvular blood flow was characterized as positive, negative, or absent (no flow) by analyzing blood cell motions across the developing valve leaflets. For embryos lacking blood cells, the plasma was labeled by injecting microbeads (Bangs Laboratories) into the yolk sac. The region of interest was defined relative to the atrium and ventricle and moved with the valve plane during the cardiac cycle. Blood flow direction was marked in every frame taken during the cardiac cycle, and the retrograde flow fraction (RFF) was determined by dividing the total number of frames exhibiting retrograde flow by the total number of frames per cycle. For each treatment, five to 15 embryos were analyzed. The boxes represented in Figures 1, 2, and 4 represent the average flow observed during a minimum of ten heartbeats.

### Shear Stress Estimates

Instantaneous blood cell velocity as a function of heart cycle time in the developing heart was assessed at 48 hpf by tracking blood cells manually in the AV canal over an average of four frames. Two heartbeats were analyzed in each condition. Shear stress was calculated as in [14]. The velocity of blood in the heart was modeled as
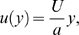
where *U* is the centerline velocity, *a* is the half-width of the region of interest (that is, the radius), and *y* is the distance from the wall. The shear stress is
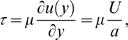
where *μ* is the dynamic viscosity of the fluid with units g·cm^−1^·s^−1^. We measured the AV canal diameter every ten frames to set *a* (on average *a* = 5 µm).

The force exerted on a cell of surface area *A* is
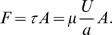



We assumed that the size of a cell in the AV canal was constant using 10 µm^2^ as its exposed surface area. The energy expenditure during one cardiac cycle (*E*, in dyne·cm) on a single cell is therefore given by
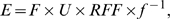
where RFF is the retrograde flow fraction and *f* is the heart rate (s^−1^).

### Hematocrit Count

Blood cells were imaged within the eye capillary. We counted the number of cells crossing a virtual line during the same time window in controls and MO-treated embryos (Video S5). Blood viscosity in *gata* morphants was estimated using the plot of relative viscosity versus particle volume fraction [52] after measurement of the particle volume fraction assuming fish blood composition is similar to that of humans.

### Lidocaine Treatment

Heart rates of experimental embryos were decreased by dosage-dependent exposure to lidocaine added to the bathing solution. Lidocaine was drawn from the stock solution (1% stock, Abbott Laboratories) and diluted into wells containing artificial pond water and approximately five embryos. Embryos were exposed to lidocaine for 24 h beginning at 31 hpf, the developmental stage marked by the transition from unidirectional to bidirectional flow. Assays of valve morphology and function were carried out at 96 hpf, a stage in which all wild-type fish hearts have at least one well-developed valve leaflet. Surviving embryos (>80%) were washed three times, placed in artificial pond water, and incubated at 28.5°C until being imaged (4 dpf). Normalized heart rates were calculated by dividing the heart rates of individuals (*n* = 30) exposed to lidocaine by the heart rates of individuals under control conditions. Heart rates were measured after 1 h of continuous exposure to lidocaine (Figure 2A).

### Temperature

Zebrafish heart rates are regulated by ambient temperature. Unless otherwise noted, embryos were incubated at 28.5°C (VWR Scientific incubator, model 2030). To increase heart rates, a higher temperature (32 or 34°C) incubator (Thermolyne, model 37900) was used. Edema and abnormal cardiogenesis were observed when embryos were raised at 16°C, 20°C, and 35°C.

### Morpholine Oligonucleotides

Two MOs targeted against the putative translational site of *klf2a* were obtained from Gene Tools LLC (5′-gtaaaatcgttccactcaaagccat-3′-MO1; 5′-agctgagatgcatggacctgtccag-3′-MO2). MOs were dissolved in 5 mM Hepes (pH 7.6) and were injected into one-cell stage embryos (total amount of 7 or 15 ng per embryo). We found that the two MOs induced the same range of malformations (valve malformation: 40%, *n* = 15 for MO1; 52%, *n* = 36 for MO2; edema: 33%, *n* = 84 for MO1; 36%, *n* = 86 for MO2). The specificity of each MO was assessed using a standard eGFP fusion approach where the eGFP sequence (pEGFP-N1, Clontech) was fused by amplifying eGFP via PCR using primers containing the target sequence of each MO and a sp6 sequence in order to translate the PCR product (mMESSAGE mMACHINE sp6, Ambion) (Figure S10). Control embryos were injected with a similar amount of a standard mismatch MO provided by Gene Tools LLC (5′-agGtgaCatgcatCgacctCtcgag-3′). The specificity of this MO was addressed using the eGFP fusion approach (Figure S11), and its effect on valve development was analyzed using *Tg(flk1:EGFP) * embryos (Figure S11). Specificity of the MOs was further assessed by analyzing the ability of *klf2a* mRNA overexpression to rescue the MO-induced edema. A total of 100 pg of mRNA was coinjected with 15 ng of each MO, and edema was scored at 32 hpf (Figure S10). MOs to *gata1*, *gata2* and *gata1/2* were used as in [34], *cx36.7* as in [15], and *myh6* as in [13].

### In Situ Hybridization

ISHs were performed as described in [53] using the following probes: *cmlc2*, *bmp4* (both provided by L. Trinh, California Institute of Technology), *notch1b* (provided by M. Lardelli, University of Adelaide), *nppa* (provided by T. Zhong, Vanderbilt Medical School), and *klf2a* probe (obtained by PCR amplification of the plasmid IRBOp991B0734D provided by RPDZ, Berlin).

### Valve Development Assay

A random sample of experimentally manipulated embryos was imaged at 96 hpf and scored based on the presence of valve leaflets. A focal plane with the atrium, ventricle, and AV canal in view was chosen to illustrate the phenotype. In cases where leaflets were difficult to identify (<2%), the presence or absence of transvalvular retrograde flow was used to determine abnormal or normal valvulogenesis, respectively.

### Cell Shape Assay

A random sample of experimentally manipulated *Tg(flk1:EGFP) *embryos was imaged at 72 hpf, and a section plan of 10 µm was made using the substack maker plugin with Image J. Cell shapes were reconstructed in three dimensions using the contour surface key in Imaris (Bitplane). A minimum of two embryos and ten cells in each condition were reconstructed. We then calculated the ratio of the length of the two longest sides and used a *Z*-test for two proportions to perform the statistical analysis.

### Real-Time RT-PCR

At 56 hpf, embryonic hearts were dissected in egg water after MO injection or lidocaine treatment. Two to three batches of ten hearts for each condition were pooled, and RNA was extracted using Trizol. RT was performed using the same amount of extracted mRNA and further tested by RT-PCR using the 96-well plate ABI 7000 QPCR machine in a SYBR Green (Bio-Rad) assay. The fold changes were calculated by the DCt method using a reference gene (zebrafish TBP) and plotted as a percentage of expression normalized to control. ANOVA tests were performed using Instat (Graphpad Software, Inc).

## Supporting Information

Figure S1 **Oscillatory flow is observed in the AV canal before valves become functional.** (A–D) Confocal scans of hearts (ventral view, anterior to the top) at four developmental stages showing the morphology of the developing heart between 36 and 120 hpf. The endocardial tissue in the AV canal at 48 hpf in shown by the arrow in (C). Valve leaflets appear at 84 hpf and are mature by 120 hpf. The black box underlines the location of blood flow analysis for each stage (A–D). Scale bar indicates 50 µm. (E–H) Transvalvular flow direction over time shows that mature valve leaflets are necessary to prevent retrograde flow in the heart. Anterograde flow from the atrium to ventricle is shown in black, retrograde flow from the ventricle to the atrium in red, and no flow between the chambers is shown in white.
(2.39 MB TIF)

Figure S2 ***klf2a*** **expression is localized to the endothelial cells of the AV canal.** (A) Brightfield image of *klf2a* mRNA distribution at 48 hpf using NBT-BCIP revelation. (B) Maximal intensity projection of 15 sections obtained by confocal microscopy (633-nm excitation wavelength) reveals the specific expression domain of *klf2a* to the innermost cell layer of the heart. (C) Profile plot of the pixel intensity measured along the bottom white line in (B) showing increased signal in the AV canal (white arrows). (D and E) Drawings locating the endothelial (e) and myocardial (m) layer on the picture. (F, I, and J) Maximal intensity projection of ten sections obtained by confocal microscopy (633-nm excitation wavelength) reveals that the specific expression domain of *klf2a* increases and becomes brighter to the innermost cell layer of the heart at during the valve elongation stage (60 hpf). (G) By comparison, expression of *cmlc2* labels the myocardium and not the endothelium. (H) Same imaging procedure using an embryo not labeled with NBT-BCIP showing no staining.
(6.54 MB TIF)

Figure S3 **Decreased blood cells number do not affects heart chamber patterning as well as head and trunk vasculogenesis.** (A, B, F, G, K, and L) *nppa* and *bmp4* expression is unaffected in *gata1* (F and G) and *gata2* (K and L) morphants compared to controls (A and B) showing that heart chambers and AV canal patterning is normal when blood cell numbers decrease. (C–E, H–J, and M–O) GFP expression in *Tg*(*flk1:EGFP)* delimitates the cardiovascular system as it is limited to every endothelial cells in the embryo (C, H, and M). Details of the head (D, I, and N) and trunk (E, J, and O) vasculature in controls (C–E), *gata1* (H–J), and *gata2* (M–O) show that no obvious malformation of the cardiovascular system is visible when blood cell number decreases. Arrows in (D, I, and N) point to the fourth branchial arch; arrows in (E, J, and O) point to secondary sprouts of the trunk cardiovascular wiring. Panels (C, H, and M) are each a composite of two original images.
(7.34 MB TIF)

Figure S4 **Quantitative analysis of the blood flow observed in the AV canal at 48 hpf.** (A) Shear stress estimated in the AV canal at 48 hpf. (B) Recapitulative table of the different flow features observed in the AV canal after the different treatments done in this paper. The energy expenditure of blood (E) required by blood cells going through the AV canal was calculated during the retrograde and anterograde flow portions of the heart cycle. It directly depends on the magnitude of the wall shear stress (WSS) and provides an estimate of the amount of WSS received by a single cell by taking into account the period of stimulation and the wall shear force intensity generated at each heart beat (see Materials and Methods). (C) Normalized flow velocity observed in *gata* morphants. (D) Outline summarizing the experimental outcome of decreasing oscillatory flow by decreasing blood viscosity (*gata1* and *gata2* MO). The color code for gene expression is the same as in Figure 2.
(2.77 MB TIF)

Figure S5 **Decreased retrograde flow via changes in contractility affects valve morphogenesis.** (A–H) Flow pattern at 48 hpf and associated confocal sections of the valve-forming region at 96 hpf in (A) control, (B) *cx36.7* (see also Video S6), (C) *myh6* (Video S6), (D) *ttna* (Video S6) knock downs, and (E) in the *silent heart* (*sih*) mutants. *myh6*, *ttna*, and *sih* inactivation leads to a dramatic decrease in the RFF and valve defects, whereas *cx36.7* knock down has an almost normal RFF and valves compared to the control. (F–H) *klf2a* expression in (F) control, (G) *cx36.7*, and (H) *myh6* morphants. Absence of *klf2a* expression was observed in *myh6* morphants (41%, *n* = 36) (H), but normal expression levels were observed in *cx36.7* morphants (75%, *n* = 50) (G). These two populations were significantly different (α = 0.1). (I) Energy expenditure comparison between control, *gata1*, and *myh6* morphants during the retrograde, anterograde, or both flow direction phases. The apparition of valve dysgenesis coincides with a low energy expenditure during phases of retrograde flow rather than a reduction of the overall energy expenditure during phases of anterograde and retrograde flow. (J) Proportionally decreased RFF through treatment with *cx36.7*, *myh6*, or *ttna* MOs leads to an increase in valve defects. The maximal effect is observed in no flow (*sih*) or no RFF (*ttna*) conditions.
(2.26 MB TIF)

Figure S6 **Strong phenotype triggered by lidocaine treatment.** (A) Control conditions (B) After treatment with lidocaine, 17% (*n* = 36) embryos do not have endothelial tissue thickening. (C and D) *bmp4* expression in (C) lidocaine-treated and (D) untreated embryos. In treated embryos, the heart tube is very immature, a situation very similar to that observed in the no-flow conditions reported in [14]. Such embryos were not used for flow analyses or qPCR, nor were they tested for valve morphogenesis at later stages. White arrow points to the AV canal.
(5.54 MB TIF)

Figure S7 ***notch1b *expression after lidocaine treatment.** *notch1b* is expressed at the AV boundary in control embryos (A and C) and after 5 h of lidocaine treatment (100%, *n* = 47; (B)) but disappears after 15 h of lidocaine treatment (61%, *n* = 36; (D)). Anterior is to the top.
(1.44 MB TIF)

Figure S8 **Expression of** ***notch1b*** **,** ***bmp4*** **, and** ***cmlc2*** **in control ([A, C, and E], respectively) and** ***klf2a*** **MO-treated ([B, D, and F], respectively) embryos.** A strong phenotype after *klf2a* MO treatment is visible in a minority fraction of embryos treated with *klf2a* MO, which display immature heart growth (13%, *n* = 20). In these strongly affected embryos, the heart tube morphology is similar to that observed in conditions were blood flow is suppressed (see [7]); they were not used for flow analysis, qPCR, or for scoring valve morphogenesis at later stages.
(1.60 MB TIF)

Figure S9 **Expression of three marker genes at 36 hpf in the heart of normal and** ***klf2a*** **morphants.** (A and B) *cmlc2* expression is essentially normal in the *klf2a* morphants, showing that chamber specification occurs independently of *klf2a*. (C and D) *bmp4* mRNA distribution at 36 hpf showing that expression is normal in the MO-treated embryo in the AV node region at that stage. (E and F) *notch1b* expression decreases in the AV boundary of the *klf2a* morphants at 36 hpf (*n* = 24, 63%; compare expression at tip of arrows). Arrows point to the AV boundary in all panels.
(2.53 MB TIF)

Figure S10 **Validation of the MO strategy.** (A–L) MOs against *klf2a* block the translation of eGFP fusion proteins carrying their target sequences. (M–X) Control MO (a mismatch of MO2) do inhibit the translation of eGFP fusion proteins carrying its target sequence (M–R), whereas MOs directed against *klf2a* cannot block the translation of the target sequence of the mismatch MO (S–X), validating the specificity of each MO.
(5.16 MB TIF)

Figure S11 **(A–E) Injection of** ***klf2a*** **mismatch morpholino does not affect valve invagination and cell shape.** (F) Overexpression of *klf2a* mRNA rescues *klf2a* MO-mediated phenotype. (A–B') Comparison of the valve phenotype between the different treatment affecting valvulogenesis using *Tg* (*flk1:egfp*) at 72 hpf. GFP is expressed in the endothelial cell layer and highlights the developing valves. (A, B, and B') control embryo, (C, D, and D') *klf2a* mismatch morphant. (B' and D') Schematic representation of the panels (B and D) outlining the endothelial cells within valve-forming region (yellow) and the heart lumen (white). Mismatch MO injection leads to a normal ingression of the endothelial cells and cuboidal cell rearrangement showing that leaflet invagination occurs properly and that there is no nonspecific effects due to MO injection. (F) Percentage of rescue obtained after overexpression of *klf2a* mRNA concomitantly with *klf2a* MO (*n* = 115 for MO1, *n* = 49 for MO2) compared with *klf2a* MO injected embryos (*n* = 84 for MO1 and *n* = 88 for MO2). A, atrium; V, ventricle.
(1.70 MB TIF)

Video S1 **Transvalvular flow changes dramatically during cardiac morphogenesis.** Heartbeats in BODIPY-ceramide-stained embryos from four developmental stages (36, 72, 84 and 120 hpf) are shown. At each stage, the age, period length, and transvalvular flow direction are shown. Valve leaflets begin to develop by 84 hpf and are mature by 120 hpf. Transvalvular retrograde flow exists until mature valve leaflets develop.
(5.58 MB MOV)

Video S2 **Three dimensional reconstruction of** ***klf2a*** **expression in the AV canal endothelium.**
(2.37 MB MOV)

Video S3 **Transvalvular flow changes in the AV canal and atrium at 36 hpf, 48 hpf, and 56 hpf in wild-type embryos (which also serve as controls [CTL]).**
(8.86 MB MOV)

Video S4 **Transvalvular flow changes in the A–V canal in** ***gata1*** **morphants,** ***gata2*** **morphants and** ***gata1/2*** **morphants at 48 hpf.** The RFF is increased in *gata1* and *gata1/2* compared to controls (see Video S3), whereas the RFF in *gata2* morphants is decreased compared to controls, *gata1*, and *gata1/2* morphants.
(8.34 MB MOV)

Video S5 **Hematocrit is severely reduced in** ***gata2*** **morphants at 48 hpf.** Blood cells traveling in an eye capillary in control (top panel in the video) and *gata2 *morphant (bottom panel of the video).
(0.60 MB MOV)

Video S6 **Transvalvular flow changes in the AV canal in cx36.7,** ***ttna*** **, and** ***myh6*** **morphants at 48 hpf.**
(9.25 MB MOV)

Video S7 **Transvalvular flow changes in the AV canal of control embryos at 3 Hz, 2.4 Hz, 1.5 Hz, and 1.2 Hz.** Wild-type hearts at 48 hpf display normal RFF when incubated at normal temperatures but display reduced RFF when incubated at elevated temperatures (which artificially increases the heart rate) and when incubated at low temperatures (which artificially decreases the heart rate).
(9.70 MB MOV)

Video S8 **Reduced oscillatory flow leads to heart valve dysgenesis.** Wild-type hearts at 96 hpf have functional valve leaflets that prevent retrograde flow across the AV valve. Fish exposed to reduced oscillatory flow through lidocaine exposure experience valve dysgenesis. Retrograde flow results from the absence of valve leaflets. Fish exposed to lidocaine and incubated at elevated temperatures, restoring the natural heart rate, do not experience valve dysgenesis.
(8.40 MB MOV)

Video S9 **Three-dimensional cell shape in the AV canal of transgenic** ***Tg*** **(** ***flk1:EGFP)*** **embryos at 72 hpf in a control embryo, in** ***gata1*** **,** ***gata2*** **, and** ***klf2a*** **morphants, and in a lidocaine-treated embryo.** GFP is expressed in the endothelial cell layer (in white) and the yellow shape highlights the cell shape of one cell in the developing valves.
(3.56 MB MOV)

## Abbreviations

AV: atrioventricular
hpf: hours postfertilization
ISH: in situ hybridization
MO: morpholine oligonucleotide
RFF: retrograde flow fraction
WSS: wall shear stress

## References

[1] Beis D, Bartman T, Jin S. W, Scott I. C, D'Amico L. A (2005). Genetic and cellular analyses of zebrafish atrioventricular cushion and valve development.. Development.

[2] Moorman A. F, Christoffels V. M (2003). Cardiac chamber formation: development, genes, and evolution.. Physiol Rev.

[3] Armstrong E. J, Bischoff J (2004). Heart valve development: endothelial cell signaling and differentiation.. Circ Res.

[4] Chi N. C, Shaw R. M, De Val S, Kang G, Jan L. Y (2008). Foxn4 directly regulates tbx2b expression and atrioventricular canal formation.. Genes Dev.

[5] Chi N. C, Shaw R. M, Jungblut B, Huisken J, Ferrer T (2008). Genetic and physiologic dissection of the vertebrate cardiac conduction system.. PLoS Biol.

[6] Bartman T, Walsh E. C, Wen K. K, McKane M, Ren J (2004). Early myocardial function affects endocardial cushion development in zebrafish.. PLoS Biol.

[7] Hurlstone A. F, Haramis A. P, Wienholds E, Begthel H, Korving J (2003). The Wnt/beta-catenin pathway regulates cardiac valve formation.. Nature.

[8] Kokubo H, Tomita-Miyagawa S, Hamada Y, Saga Y (2007). Hesr1 and Hesr2 regulate atrioventricular boundary formation in the developing heart through the repression of Tbx2.. Development.

[9] Xin M, Small E. M, van Rooij E, Qi X, Richardson J. A (2007). Essential roles of the bHLH transcription factor Hrt2 in repression of atrial gene expression and maintenance of postnatal cardiac function.. Proc Natl Acad Sci U S A.

[10] Yamada M, Revelli J. P, Eichele G, Barron M, Schwartz R. J (2000). Expression of chick Tbx-2, Tbx-3, and Tbx-5 genes during early heart development: evidence for BMP2 induction of Tbx2.. Dev Biol.

[11] Scherz P. J, Huisken J, Sahai-Hernandez P, Stainier D. Y (2008). High-speed imaging of developing heart valves reveals interplay of morphogenesis and function.. Development.

[12] Auman H. J, Coleman H, Riley H. E, Olale F, Tsai H. J (2007). Functional modulation of cardiac form through regionally confined cell shape changes.. PLoS Biol.

[13] Berdougo E, Coleman H, Lee D. H, Stainier D. Y, Yelon D (2003). Mutation of weak atrium/atrial myosin heavy chain disrupts atrial function and influences ventricular morphogenesis in zebrafish.. Development.

[14] Hove J. R, Koster R. W, Forouhar A. S, Acevedo-Bolton G, Fraser S. E (2003). Intracardiac fluid forces are an essential epigenetic factor for embryonic cardiogenesis.. Nature.

[15] Sultana N, Nag K, Hoshijima K, Laird D. W, Kawakami A (2008). Zebrafish early cardiac connexin, Cx36.7/Ecx, regulates myofibril orientation and heart morphogenesis by establishing Nkx2.5 expression.. Proc Natl Acad Sci U S A.

[16] Brooks A. R, Lelkes P. I, Rubanyi G. M (2004). Gene expression profiling of vascular endothelial cells exposed to fluid mechanical forces: relevance for focal susceptibility to atherosclerosis.. Endothelium.

[17] Hahn C, Schwartz M. A (2009). Mechanotransduction in vascular physiology and atherogenesis.. Nat Rev Mol Cell Biol.

[18] Malek A. M, Alper S. L, Izumo S (1999). Hemodynamic shear stress and its role in atherosclerosis.. JAMA.

[19] Sorescu G. P, Sykes M, Weiss D, Platt M. O, Saha A (2003). Bone morphogenic protein 4 produced in endothelial cells by oscillatory shear stress stimulates an inflammatory response.. J Biol Chem.

[20] Passerini A. G, Milsted A, Rittgers S. E (2003). Shear stress magnitude and directionality modulate growth factor gene expression in preconditioned vascular endothelial cells.. J Vasc Surg.

[21] Parmar K. M, Larman H. B, Dai G, Zhang Y, Wang E. T (2006). Integration of flow-dependent endothelial phenotypes by Kruppel-like factor 2.. J Clin Invest.

[22] Liebling M, Forouhar A. S, Wolleschensky R, Zimmermann B, Ankerhold R (2006). Rapid three-dimensional imaging and analysis of the beating embryonic heart reveals functional changes during development.. Dev Dyn.

[23] Kortschak R. D, Tamme R, Lardelli M (2001). Evolutionary analysis of vertebrate Notch genes.. Dev Genes Evol.

[24] Milan D. J, Giokas A. C, Serluca F. C, Peterson R. T, MacRae C. A (2006). Notch1b and neuregulin are required for specification of central cardiac conduction tissue.. Development.

[25] Walsh E. C, Stainier D. Y (2001). UDP-glucose dehydrogenase required for cardiac valve formation in zebrafish.. Science.

[26] Oates A. C, Pratt S. J, Vail B, Yan Y, Ho R. K (2001). The zebrafish klf gene family.. Blood.

[27] Chen J. N, van Eeden F. J, Warren K. S, Chin A, Nusslein-Volhard C (1997). Left-right pattern of cardiac BMP4 may drive asymmetry of the heart in zebrafish.. Development.

[28] Timmerman L. A, Grego-Bessa J, Raya A, Bertran E, Perez-Pomares J. M (2004). Notch promotes epithelial-mesenchymal transition during cardiac development and oncogenic transformation.. Genes Dev.

[29] Wang X. L, Fu A, Raghavakaimal S, Lee H. C (2007). Proteomic analysis of vascular endothelial cells in response to laminar shear stress.. Proteomics.

[30] McCormick S. M, Eskin S. G, McIntire L. V, Teng C. L, Lu C. M (2001). DNA microarray reveals changes in gene expression of shear stressed human umbilical vein endothelial cells.. Proc Natl Acad Sci U S A.

[31] Garcia-Cardena G, Comander J, Anderson K. R, Blackman B. R, Gimbrone M. A (2001). Biomechanical activation of vascular endothelium as a determinant of its functional phenotype.. Proc Natl Acad Sci U S A.

[32] Fung Y (1997). Biomechanics: circulation. 2nd edition..

[33] Forouhar A. S, Liebling M, Hickerson A, Nasiraei-Moghaddam A, Tsai H. J (2006). The embryonic vertebrate heart tube is a dynamic suction pump.. Science.

[34] Galloway J. L, Wingert R. A, Thisse C, Thisse B, Zon L. I (2005). Loss of gata1 but not gata2 converts erythropoiesis to myelopoiesis in zebrafish embryos.. Dev Cell.

[35] Cummins T. R (2007). Setting up for the block: the mechanism underlying lidocaine's use-dependent inhibition of sodium channels.. J Physiol.

[36] Dekker R. J, van Soest S, Fontijn R. D, Salamanca S, de Groot P. G (2002). Prolonged fluid shear stress induces a distinct set of endothelial cell genes, most specifically lung Kruppel-like factor (KLF2).. Blood.

[37] Groenendijk B. C, Hierck B. P, Vrolijk J, Baiker M, Pourquie M. J (2005). Changes in shear stress-related gene expression after experimentally altered venous return in the chicken embryo.. Circ Res.

[38] Lee J. S, Yu Q, Shin J. T, Sebzda E, Bertozzi C (2006). Klf2 is an essential regulator of vascular hemodynamic forces in vivo.. Dev Cell.

[39] Meadows S. M, Salanga M. C, Krieg P. A (2009). Kruppel-like factor 2 cooperates with the ETS family protein ERG to activate Flk1 expression during vascular development.. Development.

[40] Dekker R. J, Boon R. A, Rondaij M. G, Kragt A, Volger O. L (2006). KLF2 provokes a gene expression pattern that establishes functional quiescent differentiation of the endothelium.. Blood.

[41] Jin S. W, Beis D, Mitchell T, Chen J. N, Stainier D. Y (2005). Cellular and molecular analyses of vascular tube and lumen formation in zebrafish.. Development.

[42] Bendig G, Grimmler M, Huttner I. G, Wessels G, Dahme T (2006). Integrin-linked kinase, a novel component of the cardiac mechanical stretch sensor, controls contractility in the zebrafish heart.. Genes Dev.

[43] Kim C. H, Cho Y. S, Chun Y. S, Park J. W, Kim M. S (2002). Early expression of myocardial HIF-1alpha in response to mechanical stresses: regulation by stretch-activated channels and the phosphatidylinositol 3-kinase signaling pathway.. Circ Res.

[44] Pan J, Fukuda K, Saito M, Matsuzaki J, Kodama H (1999). Mechanical stretch activates the JAK/STAT pathway in rat cardiomyocytes.. Circ Res.

[45] Bartman T, Hove J (2005). Mechanics and function in heart morphogenesis.. Dev Dyn.

[46] Isogai S, Lawson N. D, Torrealday S, Horiguchi M, Weinstein B. M (2003). Angiogenic network formation in the developing vertebrate trunk.. Development.

[47] Groenendijk B. C, Stekelenburg-de Vos S, Vennemann P, Wladimiroff J. W, Nieuwstadt F. T (2008). The endothelin-1 pathway and the development of cardiovascular defects in the haemodynamically challenged chicken embryo.. J Vasc Res.

[48] Atkins G. B, Wang Y, Mahabeleshwar G. H, Shi H, Gao H (2008). Hemizygous deficiency of Kruppel-like factor 2 augments experimental atherosclerosis.. Circ Res.

[49] Lecuit T, Le Goff L (2007). Orchestrating size and shape during morphogenesis.. Nature.

[50] Quintin S, Gally C, Labouesse M (2008). Epithelial morphogenesis in embryos: asymmetries, motors and brakes.. Trends Genet.

[51] Lecuit T, Lenne P. F (2007). Cell surface mechanics and the control of cell shape, tissue patterns and morphogenesis.. Nat Rev Mol Cell Biol.

[52] Ethier C, Simmons C (2007). Introductory biomechanics: from cells to organisms..

[53] Alexander J, Stainier D. Y, Yelon D (1998). Screening mosaic F1 females for mutations affecting zebrafish heart induction and patterning.. Dev Genet.

