# Supplementary figures and images for: Reversing Blood Flows Act through klf2a to Ensure Normal Valvulogenesis in the Developing Heart
Source: PLoS Biol. 2009 Nov 17;7(11):e1000246. doi: 10.1371/journal.pbio.1000246 (PMC2773122; doi:10.1371/journal.pbio.1000246)

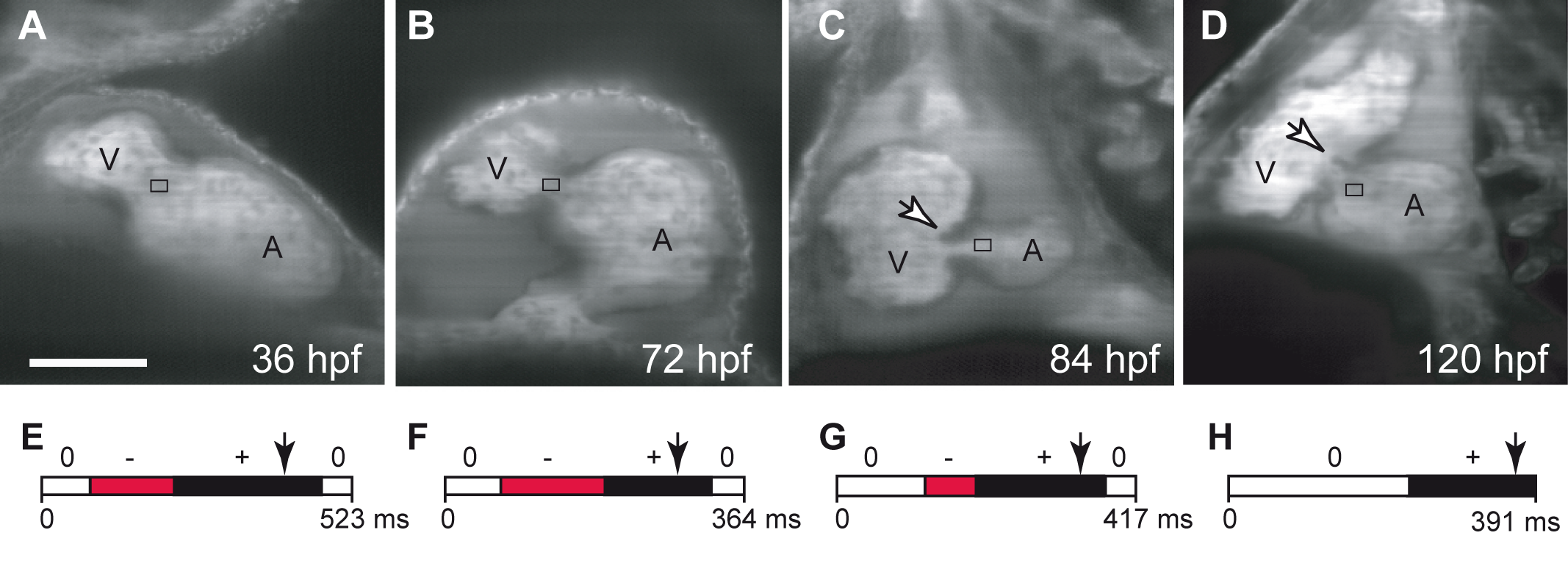

Supplement: Figure S1 — Oscillatory flow is observed in the AV canal before valves become functional. (A–D) Confocal scans of hearts (ventral view, anterior to the top) at four developmental stages showing the morphology of the developing heart between 36 and 120 hpf. The endocardial tissue in the AV canal at 48 hpf in shown by the arrow in (C). Valve leaflets appear at 84 hpf and are mature by 120 hpf. The black box underlines the location of blood flow analysis for each stage (A–D). Scale bar indicates 50 µm. (E–H) Transvalvular flow direction over time shows that mature valve leaflets are necessary to prevent retrograde flow in the heart. Anterograde flow from the atrium to ventricle is shown in black, retrograde flow from the ventricle to the atrium in red, and no flow between the chambers is shown in white. (2.39 MB TIF) [file pbio.1000246.s001.tif]

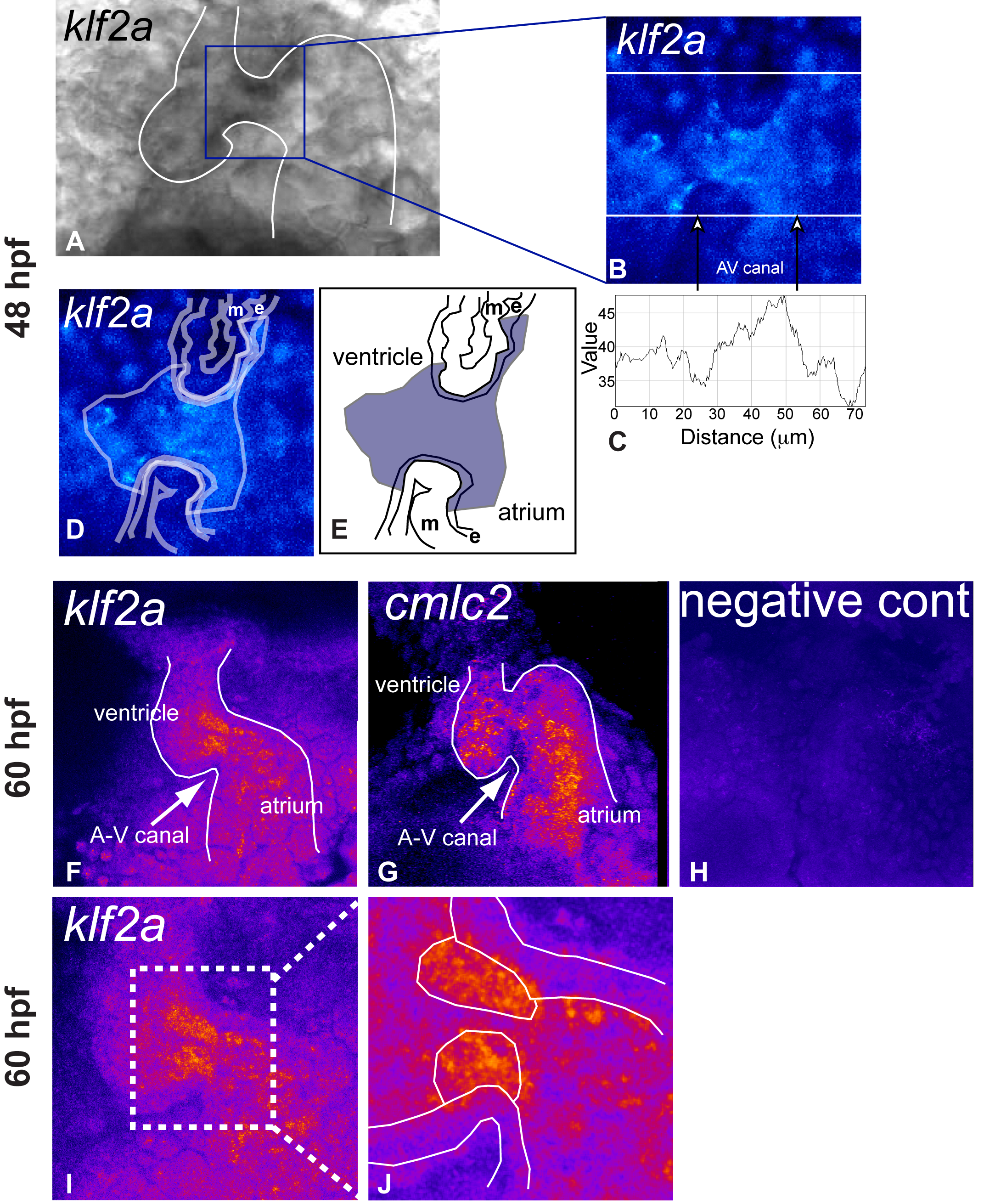

Supplement: Figure S2 — klf2a expression is localized to the endothelial cells of the AV canal. (A) Brightfield image of klf2a mRNA distribution at 48 hpf using NBT-BCIP revelation. (B) Maximal intensity projection of 15 sections obtained by confocal microscopy (633-nm excitation wavelength) reveals the specific expression domain of klf2a to the innermost cell layer of the heart. (C) Profile plot of the pixel intensity measured along the bottom white line in (B) showing increased signal in the AV canal (white arrows). (D and E) Drawings locating the endothelial (e) and myocardial (m) layer on the picture. (F, I, and J) Maximal intensity projection of ten sections obtained by confocal microscopy (633-nm excitation wavelength) reveals that the specific expression domain of klf2a increases and becomes brighter to the innermost cell layer of the heart at during the valve elongation stage (60 hpf). (G) By comparison, expression of cmlc2 labels the myocardium and not the endothelium. (H) Same imaging procedure using an embryo not labeled with NBT-BCIP showing no staining. (6.54 MB TIF) [file pbio.1000246.s002.tif]

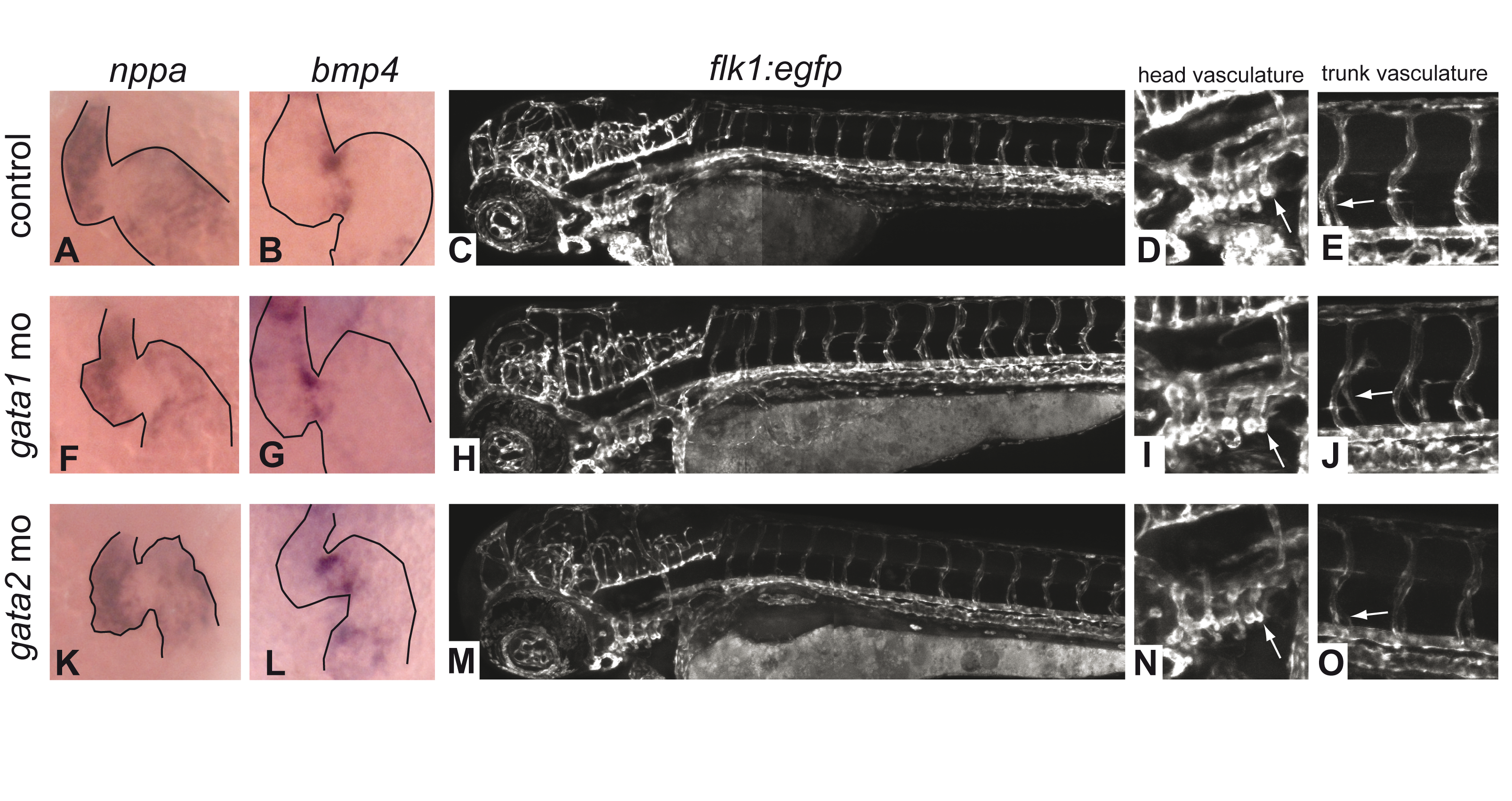

Supplement: Figure S3 — Decreased blood cells number do not affects heart chamber patterning as well as head and trunk vasculogenesis. (A, B, F, G, K, and L) nppa and bmp4 expression is unaffected in gata1 (F and G) and gata2 (K and L) morphants compared to controls (A and B) showing that heart chambers and AV canal patterning is normal when blood cell numbers decrease. (C–E, H–J, and M–O) GFP expression in Tg(flk1:EGFP) delimitates the cardiovascular system as it is limited to every endothelial cells in the embryo (C, H, and M). Details of the head (D, I, and N) and trunk (E, J, and O) vasculature in controls (C–E), gata1 (H–J), and gata2 (M–O) show that no obvious malformation of the cardiovascular system is visible when blood cell number decreases. Arrows in (D, I, and N) point to the fourth branchial arch; arrows in (E, J, and O) point to secondary sprouts of the trunk cardiovascular wiring. Panels (C, H, and M) are each a composite of two original images. (7.34 MB TIF) [file pbio.1000246.s003.tif]

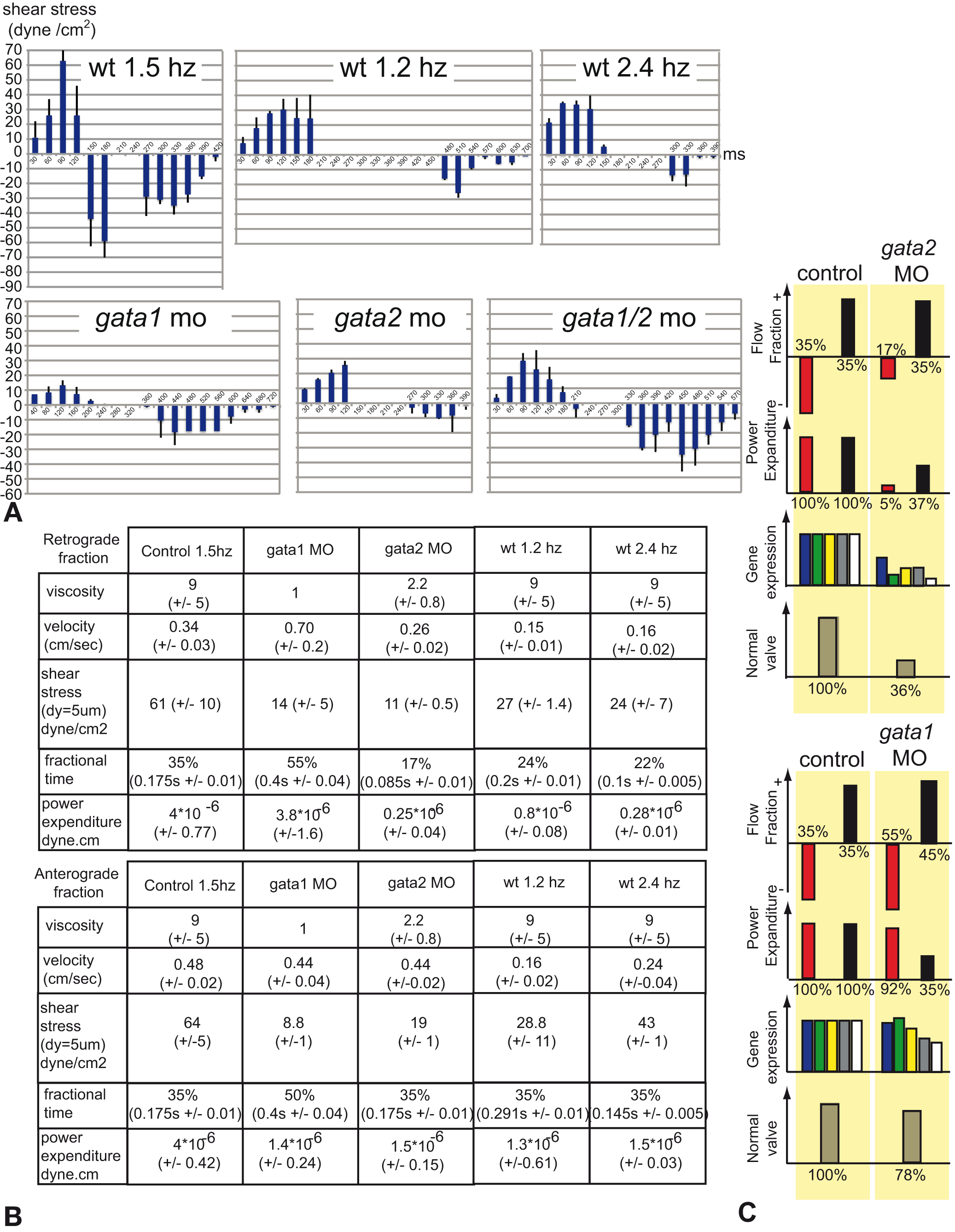

Supplement: Figure S4 — Quantitative analysis of the blood flow observed in the AV canal at 48 hpf. (A) Shear stress estimated in the AV canal at 48 hpf. (B) Recapitulative table of the different flow features observed in the AV canal after the different treatments done in this paper. The energy expenditure of blood (E) required by blood cells going through the AV canal was calculated during the retrograde and anterograde flow portions of the heart cycle. It directly depends on the magnitude of the wall shear stress (WSS) and provides an estimate of the amount of WSS received by a single cell by taking into account the period of stimulation and the wall shear force intensity generated at each heart beat (see Materials and Methods). (C) Normalized flow velocity observed in gata morphants. (D) Outline summarizing the experimental outcome of decreasing oscillatory flow by decreasing blood viscosity (gata1 and gata2 MO). The color code for gene expression is the same as in Figure 2. (2.77 MB TIF) [file pbio.1000246.s004.tif]

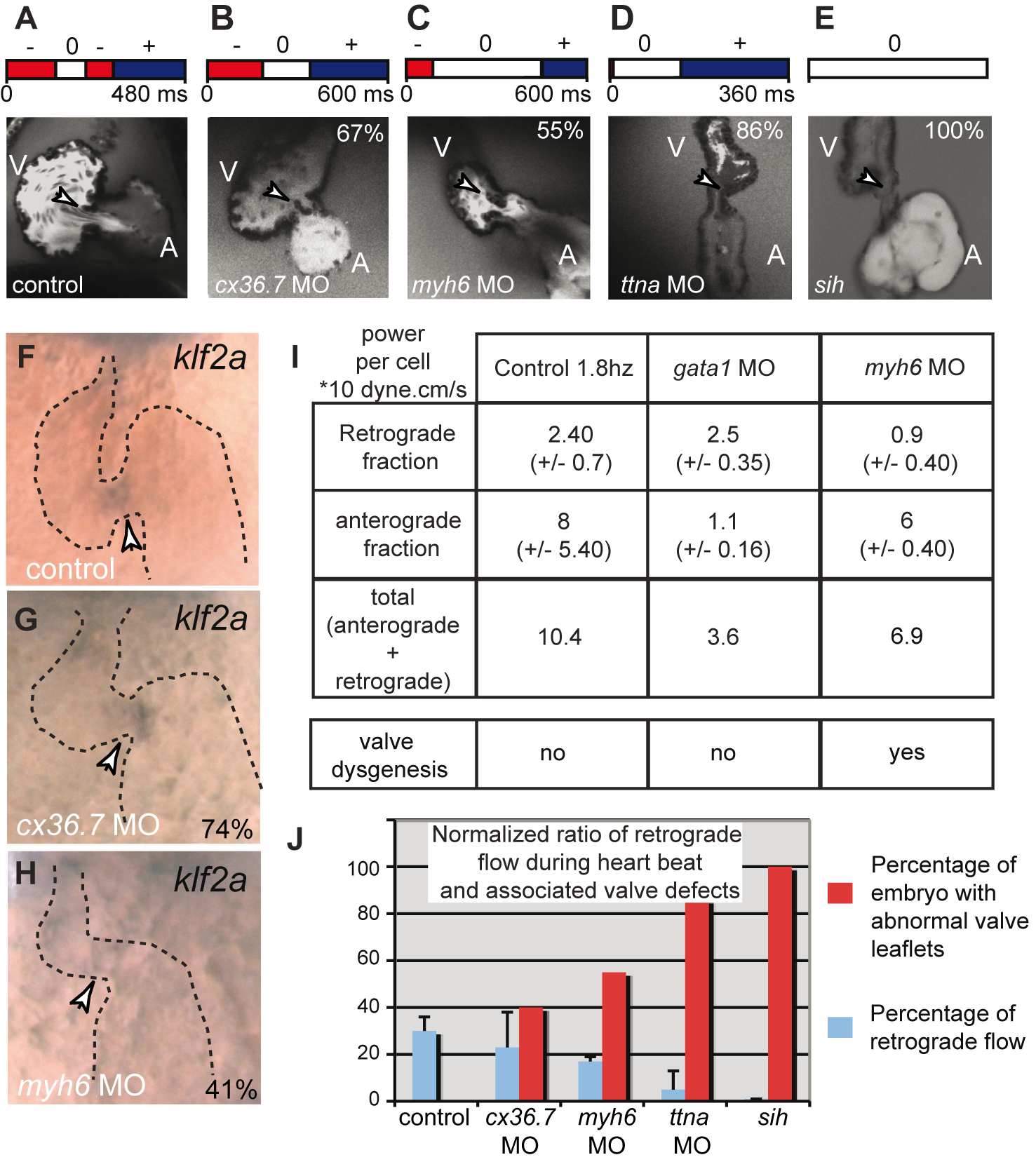

Supplement: Figure S5 — Decreased retrograde flow via changes in contractility affects valve morphogenesis. (A–H) Flow pattern at 48 hpf and associated confocal sections of the valve-forming region at 96 hpf in (A) control, (B) cx36.7 (see also Video S6), (C) myh6 (Video S6), (D) ttna (Video S6) knock downs, and (E) in the silent heart (sih) mutants. myh6, ttna, and sih inactivation leads to a dramatic decrease in the RFF and valve defects, whereas cx36.7 knock down has an almost normal RFF and valves compared to the control. (F–H) klf2a expression in (F) control, (G) cx36.7, and (H) myh6 morphants. Absence of klf2a expression was observed in myh6 morphants (41%, n = 36) (H), but normal expression levels were observed in cx36.7 morphants (75%, n = 50) (G). These two populations were significantly different (α = 0.1). (I) Energy expenditure comparison between control, gata1, and myh6 morphants during the retrograde, anterograde, or both flow direction phases. The apparition of valve dysgenesis coincides with a low energy expenditure during phases of retrograde flow rather than a reduction of the overall energy expenditure during phases of anterograde and retrograde flow. (J) Proportionally decreased RFF through treatment with cx36.7, myh6, or ttna MOs leads to an increase in valve defects. The maximal effect is observed in no flow (sih) or no RFF (ttna) conditions. (2.26 MB TIF) [file pbio.1000246.s005.tif]

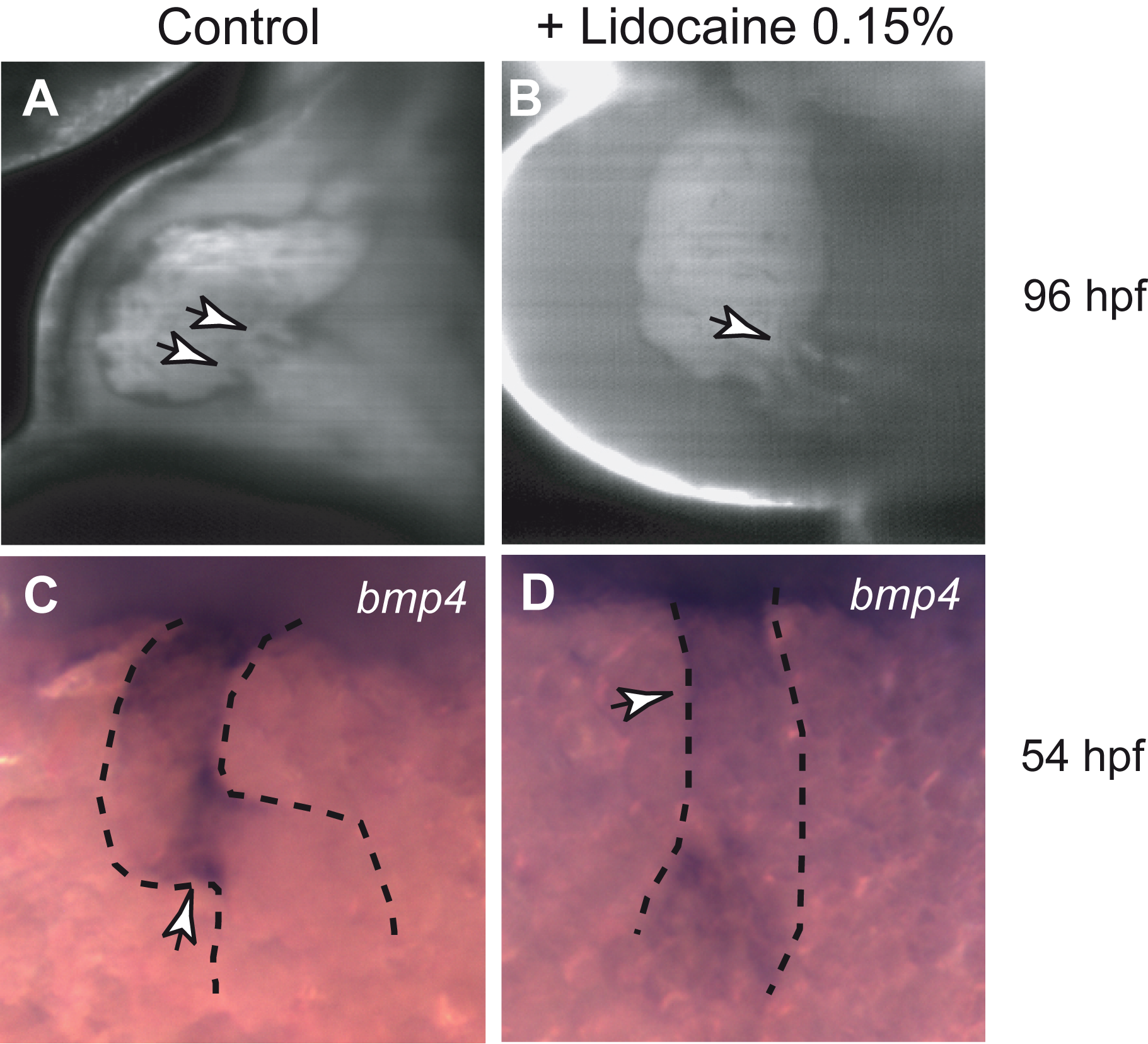

Supplement: Figure S6 — Strong phenotype triggered by lidocaine treatment. (A) Control conditions (B) After treatment with lidocaine, 17% (n = 36) embryos do not have endothelial tissue thickening. (C and D) bmp4 expression in (C) lidocaine-treated and (D) untreated embryos. In treated embryos, the heart tube is very immature, a situation very similar to that observed in the no-flow conditions reported in [14]. Such embryos were not used for flow analyses or qPCR, nor were they tested for valve morphogenesis at later stages. White arrow points to the AV canal. (5.54 MB TIF) [file pbio.1000246.s006.tif]

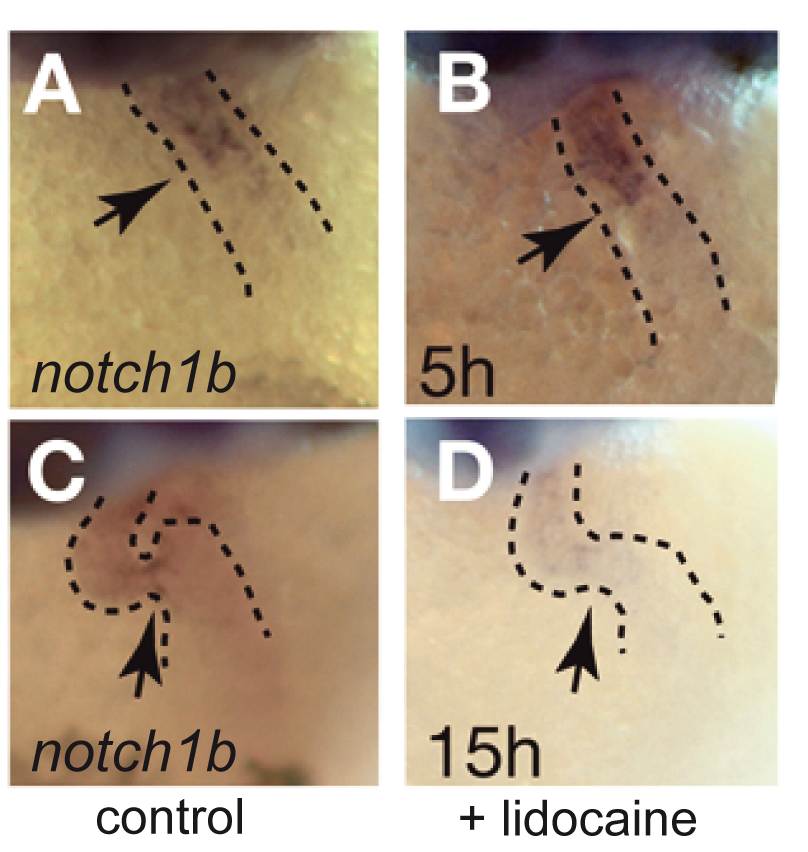

Supplement: Figure S7 — notch1b expression after lidocaine treatment. notch1b is expressed at the AV boundary in control embryos (A and C) and after 5 h of lidocaine treatment (100%, n = 47; (B)) but disappears after 15 h of lidocaine treatment (61%, n = 36; (D)). Anterior is to the top. (1.44 MB TIF) [file pbio.1000246.s007.tif]

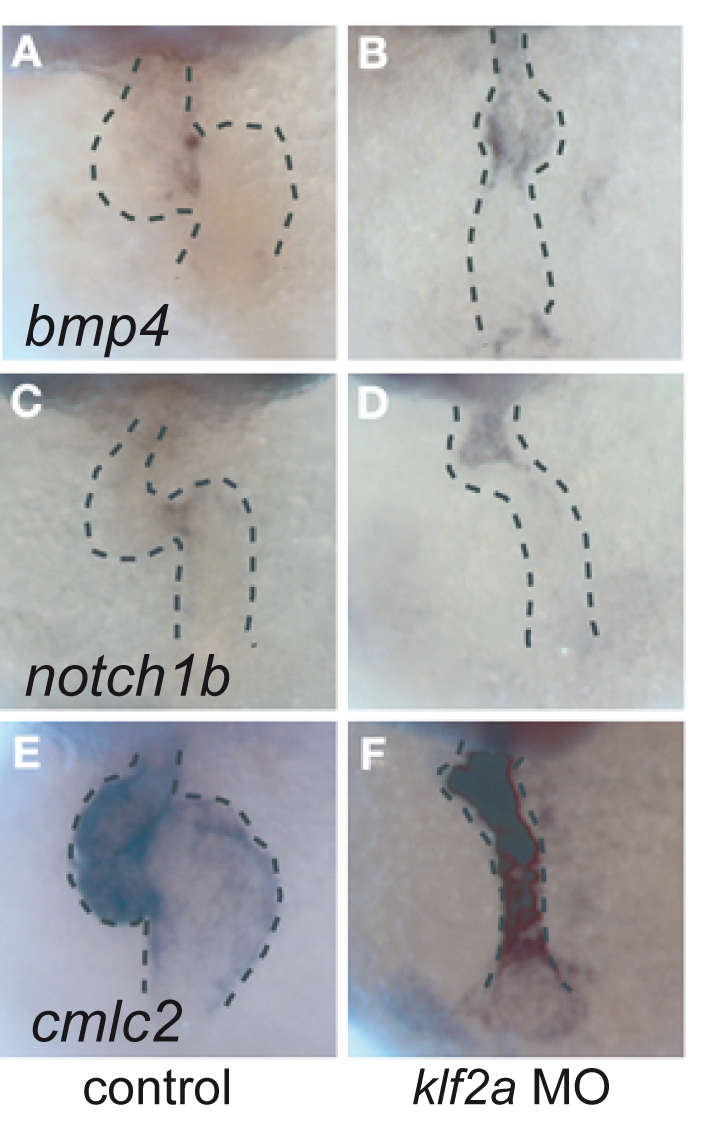

Supplement: Figure S8 — Expression of notch1b , bmp4 , and cmlc2 in control ([A, C, and E], respectively) and klf2a MO-treated ([B, D, and F], respectively) embryos. A strong phenotype after klf2a MO treatment is visible in a minority fraction of embryos treated with klf2a MO, which display immature heart growth (13%, n = 20). In these strongly affected embryos, the heart tube morphology is similar to that observed in conditions were blood flow is suppressed (see [7]); they were not used for flow analysis, qPCR, or for scoring valve morphogenesis at later stages. (1.60 MB TIF) [file pbio.1000246.s008.tif]

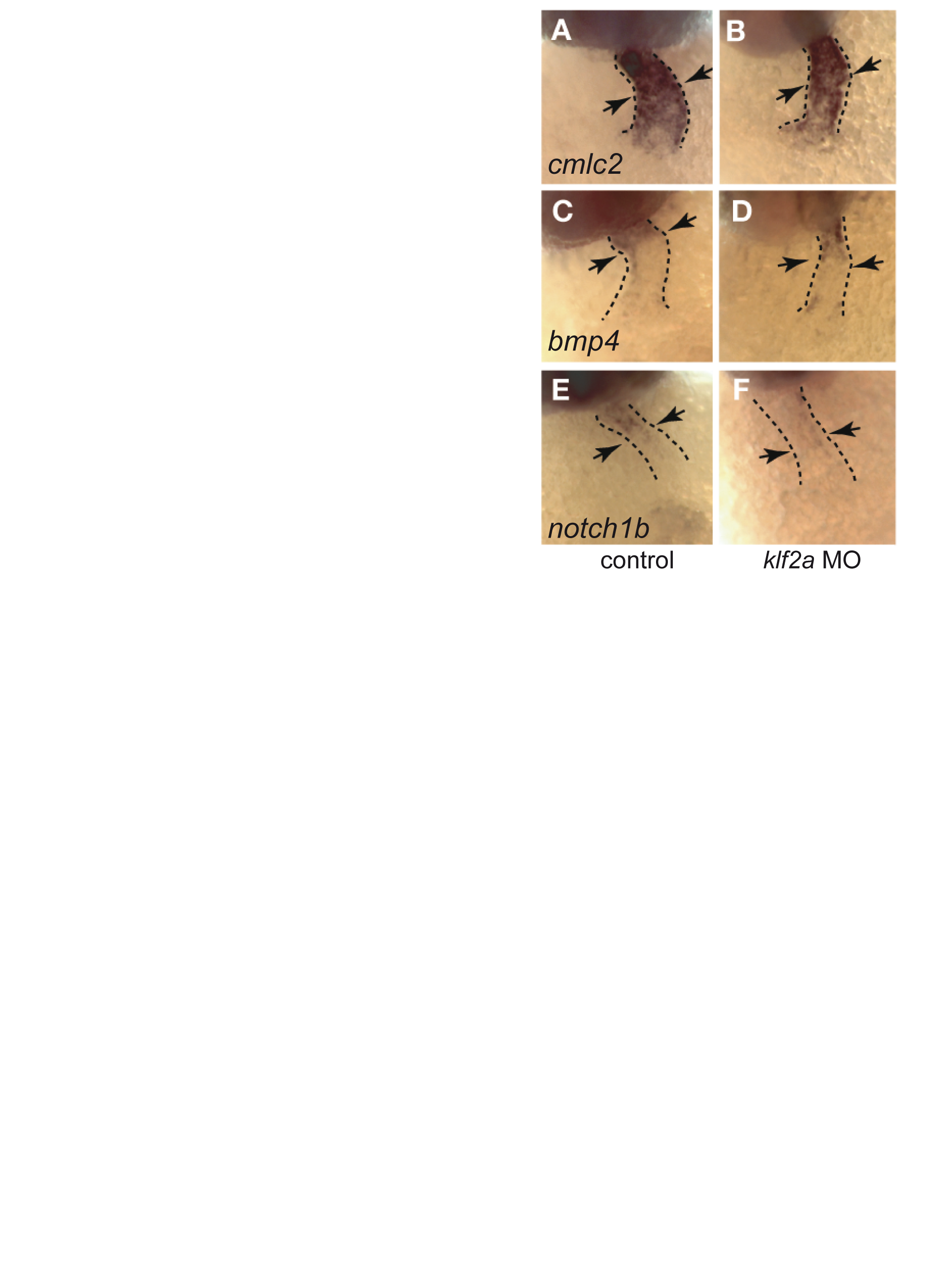

Supplement: Figure S9 — Expression of three marker genes at 36 hpf in the heart of normal and klf2a morphants. (A and B) cmlc2 expression is essentially normal in the klf2a morphants, showing that chamber specification occurs independently of klf2a. (C and D) bmp4 mRNA distribution at 36 hpf showing that expression is normal in the MO-treated embryo in the AV node region at that stage. (E and F) notch1b expression decreases in the AV boundary of the klf2a morphants at 36 hpf (n = 24, 63%; compare expression at tip of arrows). Arrows point to the AV boundary in all panels. (2.53 MB TIF) [file pbio.1000246.s009.tif]

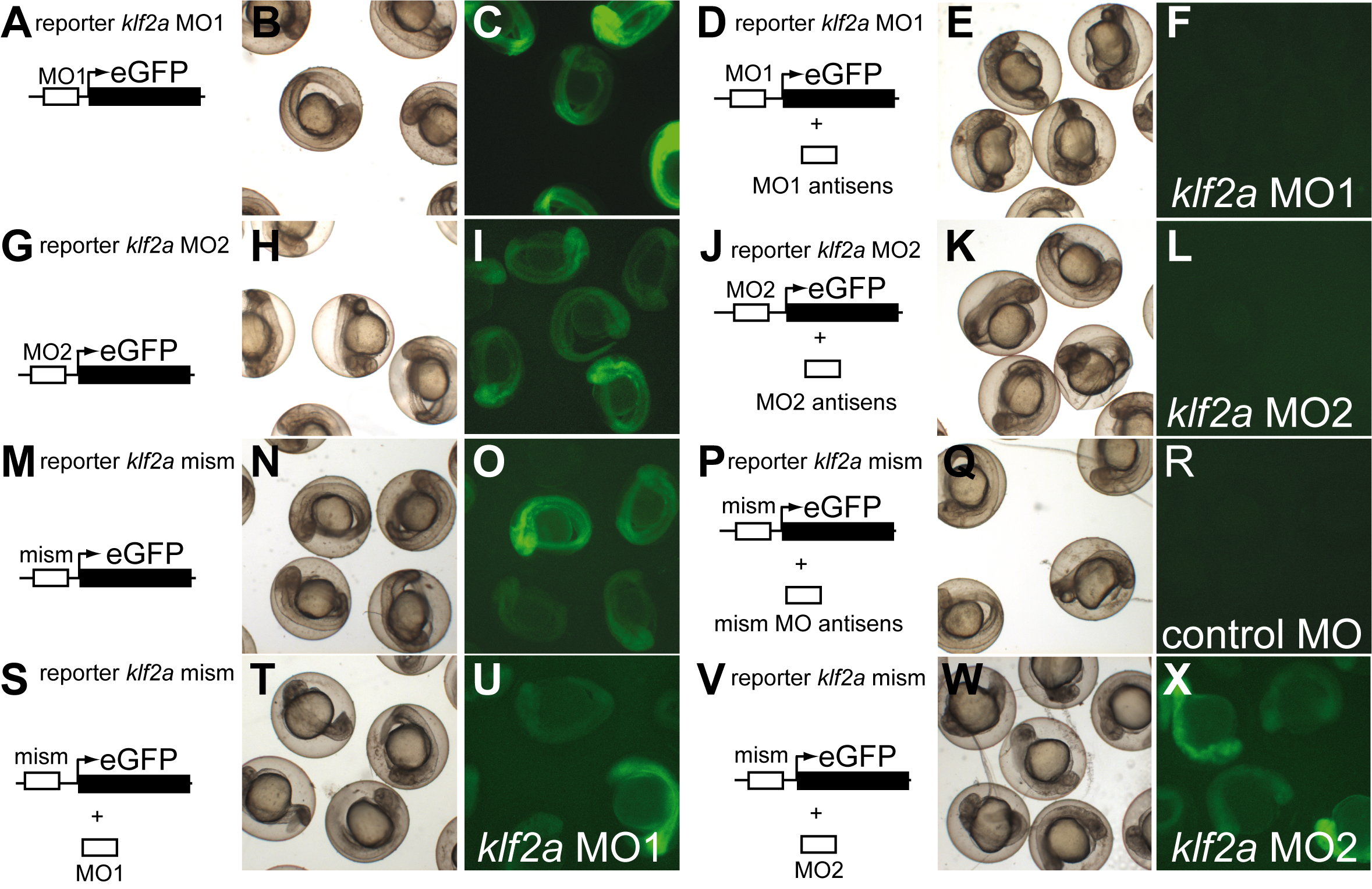

Supplement: Figure S10 — Validation of the MO strategy. (A–L) MOs against klf2a block the translation of eGFP fusion proteins carrying their target sequences. (M–X) Control MO (a mismatch of MO2) do inhibit the translation of eGFP fusion proteins carrying its target sequence (M–R), whereas MOs directed against klf2a cannot block the translation of the target sequence of the mismatch MO (S–X), validating the specificity of each MO. (5.16 MB TIF) [file pbio.1000246.s010.tif]

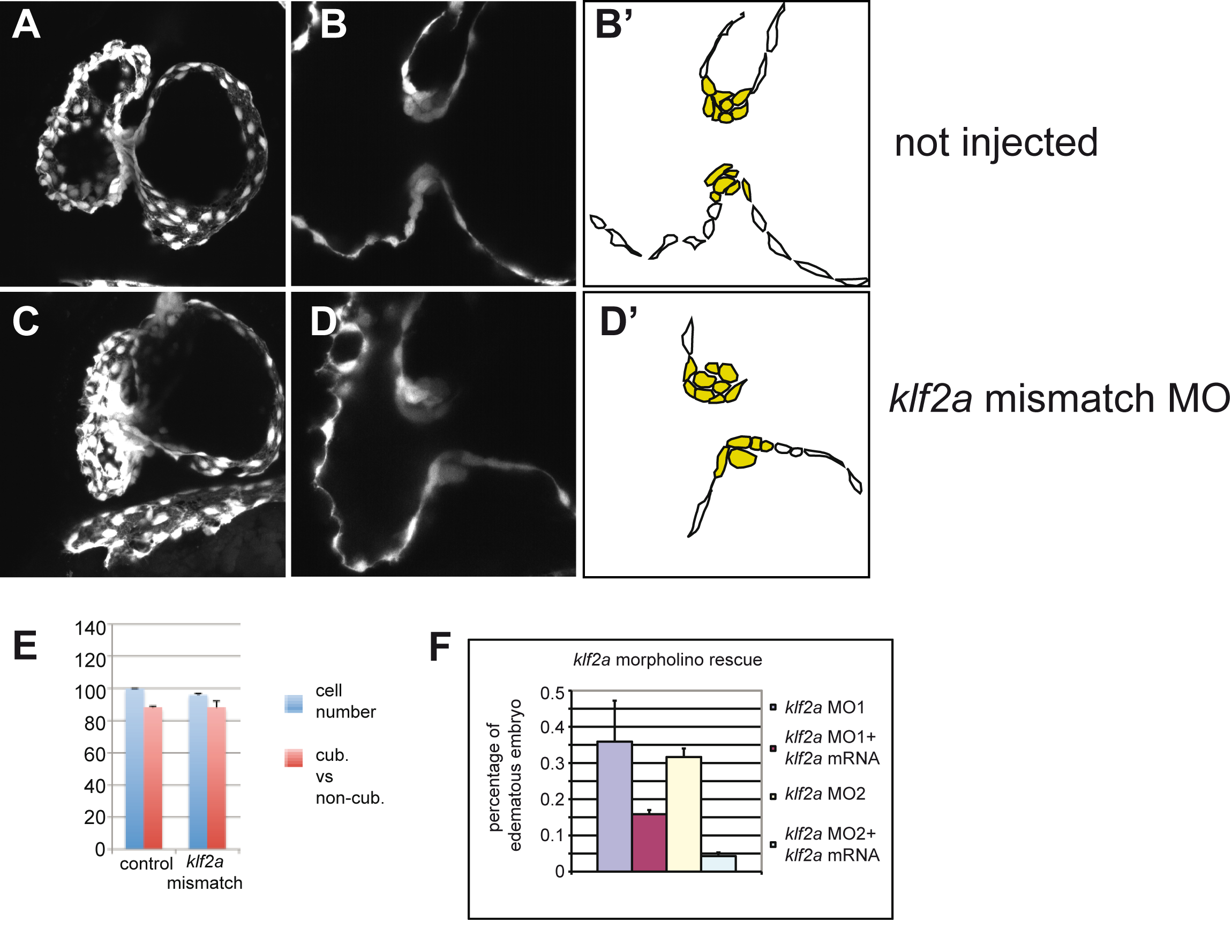

Supplement: Figure S11 — (A–E) Injection of klf2a mismatch morpholino does not affect valve invagination and cell shape. (F) Overexpression of klf2a mRNA rescues klf2a MO-mediated phenotype. (A–B') Comparison of the valve phenotype between the different treatment affecting valvulogenesis using Tg (flk1:egfp) at 72 hpf. GFP is expressed in the endothelial cell layer and highlights the developing valves. (A, B, and B') control embryo, (C, D, and D') klf2a mismatch morphant. (B' and D') Schematic representation of the panels (B and D) outlining the endothelial cells within valve-forming region (yellow) and the heart lumen (white). Mismatch MO injection leads to a normal ingression of the endothelial cells and cuboidal cell rearrangement showing that leaflet invagination occurs properly and that there is no nonspecific effects due to MO injection. (F) Percentage of rescue obtained after overexpression of klf2a mRNA concomitantly with klf2a MO (n = 115 for MO1, n = 49 for MO2) compared with klf2a MO injected embryos (n = 84 for MO1 and n = 88 for MO2). A, atrium; V, ventricle. (1.70 MB TIF) [file pbio.1000246.s011.tif]
